# Bioorthogonal Self-Immolative Linker Based on Grob Fragmentation

**DOI:** 10.1021/acs.orglett.1c03299

**Published:** 2021-10-25

**Authors:** Xhenti Ferhati, Marina Salas-Cubero, Pablo Garrido, Josune García-Sanmartín, Ana Guerreiro, Alberto Avenoza, Jesús H. Busto, Jesús M. Peregrina, Alfredo Martínez, Ester Jiménez-Moreno, Gonçalo J. L. Bernardes, Francisco Corzana

**Affiliations:** † Departamento de Química, Centro de Investigación en Síntesis Química, Universidad de La Rioja, 26006 Logroño, La Rioja, Spain; ‡ Angiogenesis Group, Oncology Area, Center for Biomedical Research of La Rioja (CIBIR), 26006 Logroño, Spain; § Instituto de Medicina Molecular Joao Lobo Antunes, Faculdade de Medicina de Universidad de Lisboa, 1649-028 Lisboa, Portugal; ⊥ Yusuf Hamied Department of Chemistry, University of Cambridge, Lensfield Road, Cambridge CB2 1EW, United Kingdom

## Abstract

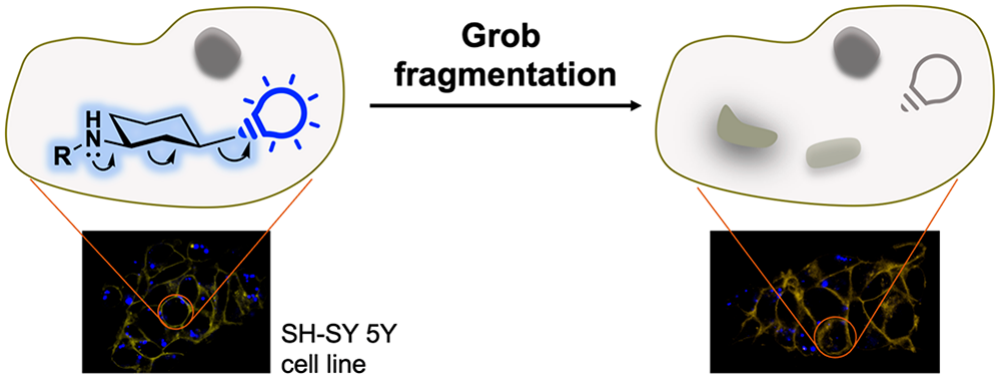

A self-immolative bioorthogonal conditionally cleavable linker based on Grob fragmentation is described. It is derived from 1,3-aminocyclohexanols and allows the release of sulfonate-containing compounds in aqueous media. Modulation of the amine p*K*_a_ promotes fragmentation even at slightly acidic pH, a common feature of several tumor environments. The Grob fragmentation can also occur under physiological conditions in living cells, highlighting the potential bioorthogonal applicability of this reaction.

Antibody–drug conjugates (ADCs) are attracting considerable attention due to their high therapeutic potential in cancer treatment.^1^ ADCs combine the high selectivity of a monoclonal antibody for a specific target on cancer cells with the toxicity of a drug attached through a linker. Once the ADC has reached its target, the payload can be released. Despite extensive research in ADCs, improvement is still highly desirable, particularly in those related to conditionally activable linker technologies. Two main families of linkers have been developed. “Noncleavable linkers” rely on proteolytic degradation of the antibody upon internalization to release the drug. On the contrary, “cleavable linkers” are designed to release the drug within or in the vicinity of the tumor cell upon a trigger stimulus. In recent years, there have been multiple reports of self-immolative linkers able to self-degrade in a spontaneous and irreversible manner through a cascade-elimination process. This process is, in general, driven by an entropy increase and the formation of thermodynamically stable products. Control of drug release is achieved by a stimulus such as an enzymatic cleavage of the linker that activates the self-immolative process.^2−11^

The most validated self-immolative linker uses a *p*-amino benzyl carbamate either coupled to a valine-citrulline (Val-Cit-PAB) or β-glucuronide, which release the cytotoxic payload upon cathepsin B or β-glucuronidase cleavage, respectively.^12^ These linkers have been successfully translated into the clinic; for example, Adcetris has been approved for the treatment of refractory Hodgkin’s lymphoma.^13^ In the last years, a range of self-immolative linkers have also been developed such as *p*-aminobenzyl ethers,^14^ cinnamyl ethers,^15^ or cyclization driven linkers^16,17^ (Figure 1a).

**Figure 1:**
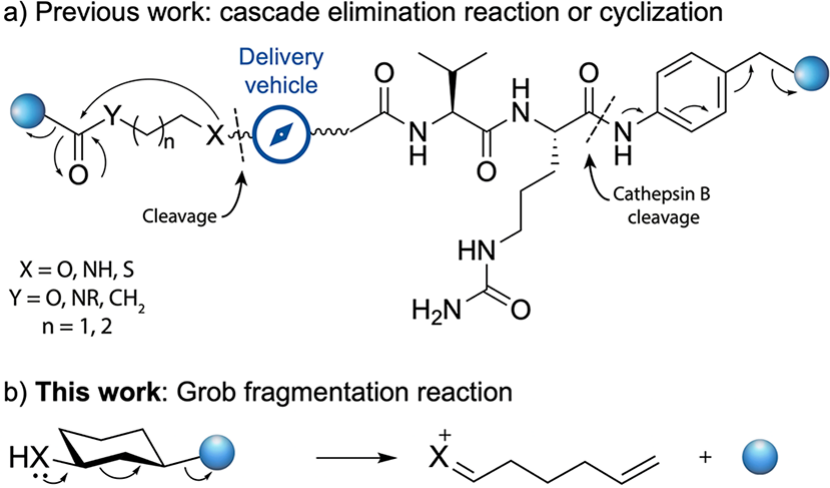
(a) Examples of reported self-immolative linkers. (b) Self-immolative linker based on Grob fragmentation.

Here, we decided to explore the Grob fragmentation^18−20^ in the design of self-immolative linkers. Substrates that can undergo Grob fragmentation are 1,3-disubstituted chains bearing a nucleophile with a negative charge or a lone electron pair at position 1, such as a heteroatom (X in Figure 1b), and a leaving group in position 3, such as halogens, sulfonates, or quaternary ammonium salts (represented as a blue sphere in Figure 1b).^19^ This fragmentation has been widely used in the synthesis of highly demanding macrocyclic structures^21^ and natural products.^22^ Typical conditions use organic solvents, high temperatures, and strong bases. Interestingly, isolated reports have used water as a cosolvent in this reaction,^23,24^ which led us to envision that the Grob fragmentation may be performed under milder conditions found in biological environments.

To this purpose, we synthesized compounds **1**–**3** shown in Figure 2. A secondary amine was chosen as a pushing group because its p*K*_a_, and thus, the corresponding reaction rate can be modulated by the installation of different substituents. Similarly, sulfonates were selected as leaving groups due to their broad presence in organic molecules with biological applications such as fluorophores. Compound **3** was used as a negative control since the nitrogen lone pair is not available and precludes the Grob fragmentation. This compound is completely stable in a mixture of CD_3_CN/PBS (1:1) buffer with a pH of 7.4 for 48 h at 37 °C, as shown by ^1^H NMR spectroscopy (Figure S7). The addition of an organic solvent to the buffer is required for the complete solubilization of these compounds.

**Figure 2:**
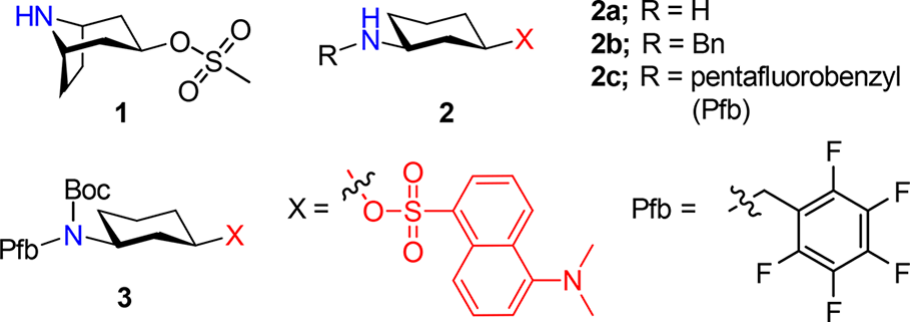
Substrates synthesized in this work to study the Grob fragmentation.

Compound **1** was also stable in a mixture CH_3_OH/PBS buffer (pH 7.4) (1:1). However, the fragmentation reaction took place when the pH of the buffer was increased from 7.4 to 8.0. In this case, a mixture of the starting material and the fragmentation product was observed by ^1^H NMR (Figure S1). The reaction was complete after 18 h when NaP_i_ buffer (pH 9.3) was used (Scheme 1 and Figure S1). These results confirmed that the Grob fragmentation can proceed in an aqueous environment in a pH-dependent manner. To investigate this property, we carried out the Grob fragmentation using compounds **2a**–**c**. It is known that the p*K*_a_ value of benzylamine is one unit lower (p*K*_a_ = 9.3 at 25 °C) than that of methylamine (p*K*_a_ = 10.7 at 25 °C).^25^ Therefore, compound **2b** is expected to have a lower p*K*_a_ than derivative **2a**. Moreover, the installation of a pentafluorinated benzyl group is expected to have a greater effect on the p*K*_a_ due to the electronegative nature of the fluorine atoms, significantly decreasing its value, and allowing for reaction to occur at slightly acidic pH (see below). We studied the Grob fragmentation in these substrates in mixtures CD_3_CN/PBS buffer (pH 7.4) (1:1) by ^1^H NMR spectroscopy. While compound **2a** did not undergo Grob fragmentation after 48 h (Figure S2), the reactions of **2b** and **2c** showed ^1^H NMR peaks in the range of 5–6.5 and around 10 ppm, corresponding to the terminal alkene and the aldehyde resulting from the hydrolysis of the imine, respectively (Scheme 1, Figures 3 and S3). According to the ^1^H NMR experiments, 69% and 61% of the dansyl sulfonate was released for compound **2c** and **2b**, respectively, after 48 h. As observed with derivative **1**, higher pH values of the buffer led to an increase in the reaction rate and yield (Figure S4).

**Scheme 1:**
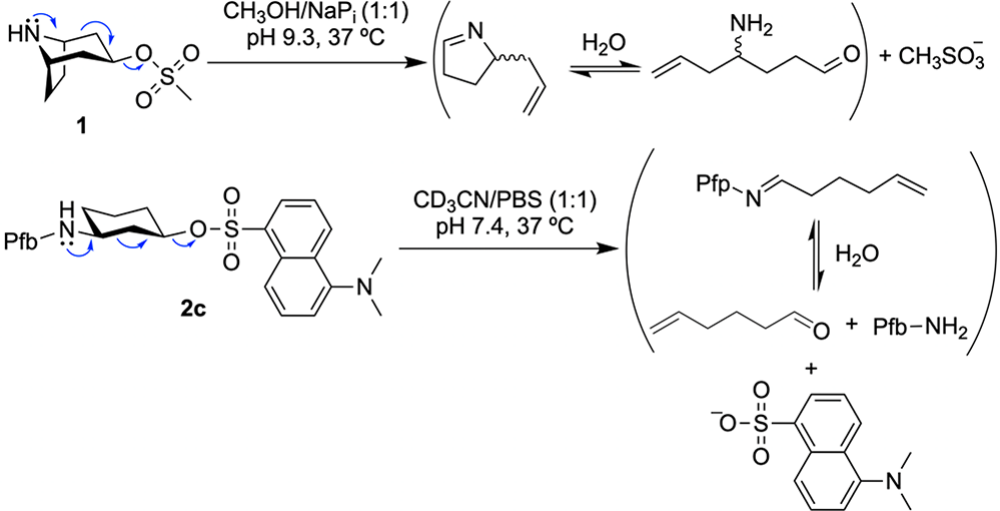
Grob Fragmentation of Compounds **1** and **2c**

**Figure 3:**
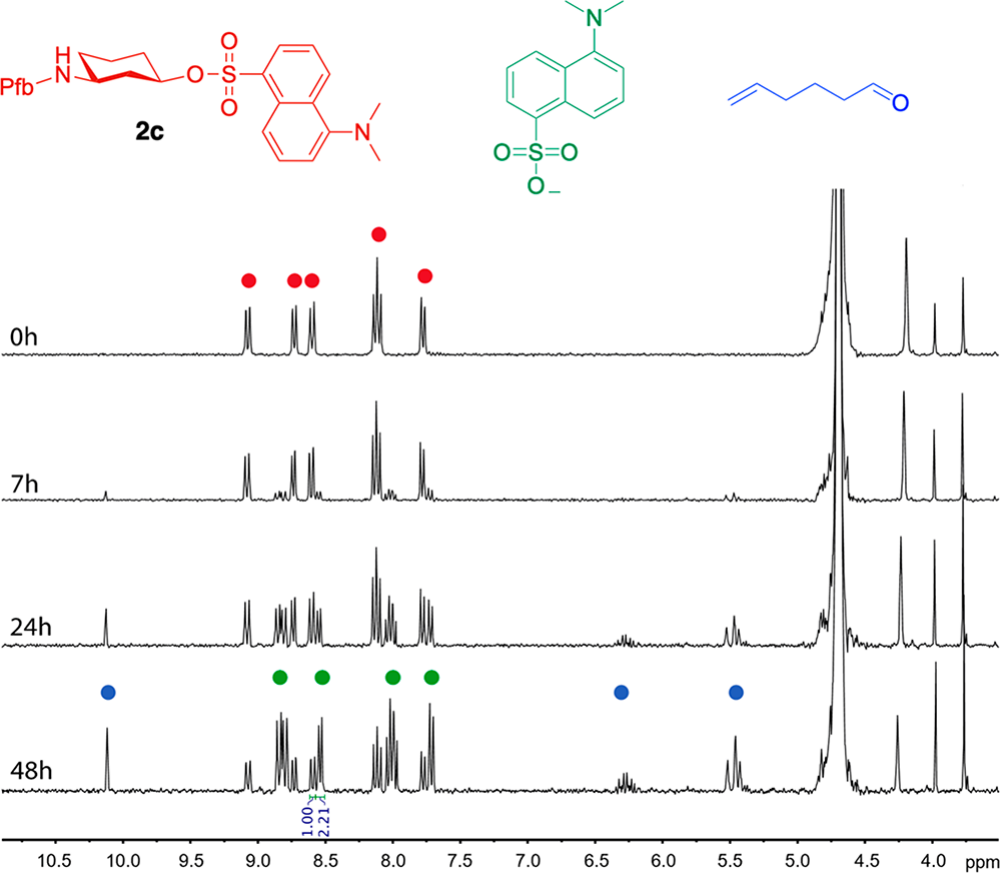
Monitoring the Grob fragmentation of **2c** by ^1^H NMR spectroscopy. The reaction was performed at 5 mM of **2c** in CD_3_CN/PBS pH 7.4 (1:1) at 37 °C.

Next, we examined the fragmentation reaction under slightly acidic pH conditions (NaP_i_ 0.1M, pH 6.0), which is a common feature of the various tumor environments.^26^ As expected, the reaction rate was dampened, but significant release of dansyl sulfonate was observed in **2b** and **2c** under these conditions after 48 h (51% and 69%, respectively, Figures S5 and S6).

Interestingly, when dansyl acid is conjugated to our Grob fragmentation scaffolds, the absorbance of this group suffers a red shift, and the absorbance maximum shifts from 312 nm for the free acid to ∼348 nm for derivatives **2c** and **3** (Figure 4a). When the latter molecules are excited at 380 nm, they emit fluorescence while free dansyl acid does not. This property was exploited to investigate the potential bioorthogonality of the reaction under physiological conditions. First, we incubated **2c** and **3** with cell medium at pH 7.5. As shown in Figure 4b, the fluorescence of compound **3** was retained after 28 h. A similar result was obtained at pH 6.0 (Figure S8), indicating that these acidic conditions are not strong enough to cleave the Boc and prompt the corresponding fragmentation. The reaction under strongly acidic conditions could favor the cleavage of the carbamate. At the same time, however, these conditions may reduce (as shown above) or hinder the rate of fragmentation since the amino group is likely to remain in the fully protonated form. On the contrary, the fluorescence decreases significantly for compound **2c** at pH 6.0 and 7.5 after 4 h (Figures 4b and S8), indicating that fragmentation of this molecule takes place successfully under biological conditions, in agreement with our previous NMR study.

**Figure 4:**
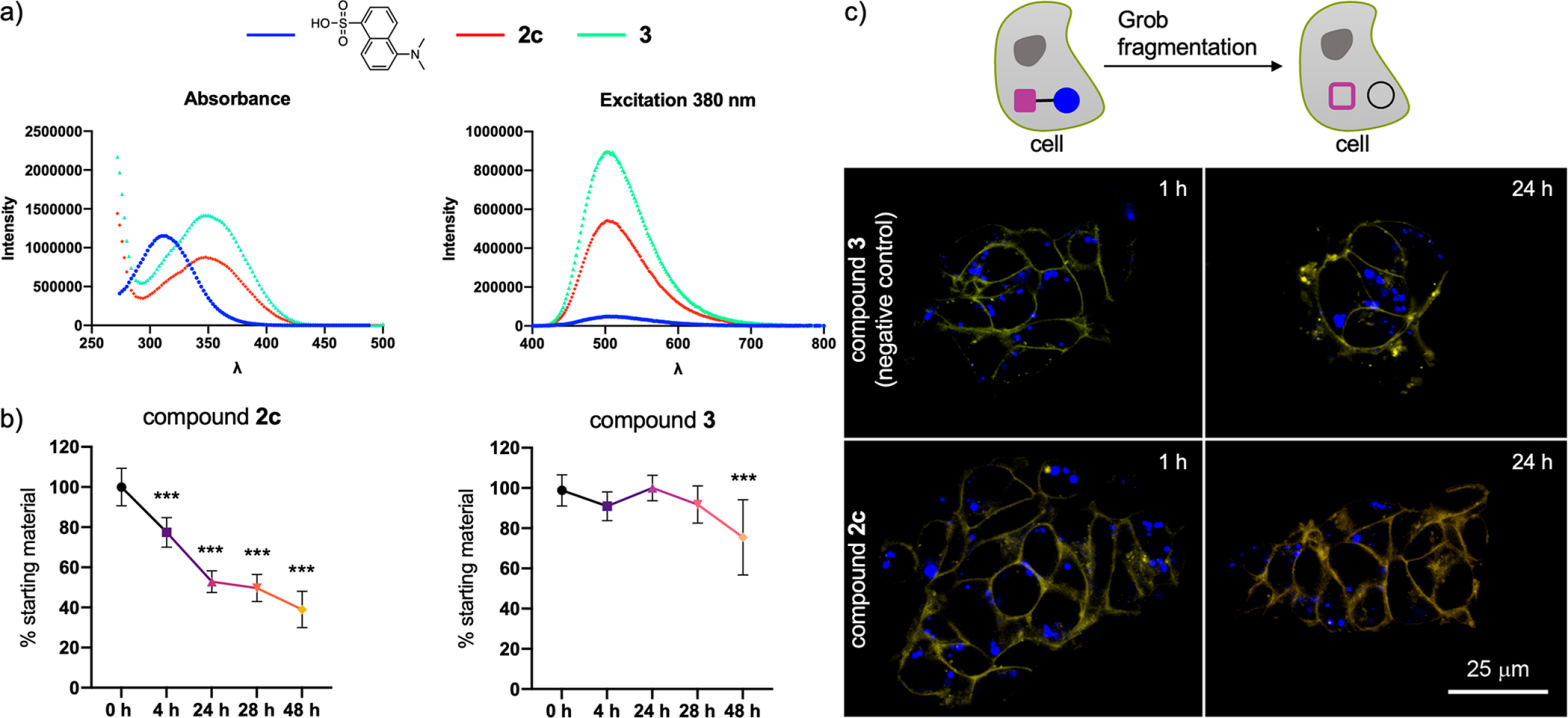
(a) Absorbance and excitation (380 nm) spectra for dansyl–OH and compounds **2c** and **3**. (b) Evolution of Grob fragmentation for compounds **2c** and **3** (20 μM) in cell medium (pH 7.5). (c) Compounds **2c** and **3** generate a fluorescent signal when irradiated in the violet spectrum (excitation 405 nm, emission 420–470 nm) that can be detected by the confocal microscope. For compound **2c**, the Grob reaction generates two fragments that produce no signal in this region of the spectrum (top). Representative confocal microscopy photographs of cell line SH-SY 5Y exposed to compounds **2c** and **3** for 1 and 24 h (bottom). The cell limit was labeled with CellMask Orange (orange color). The compounds form intracellular vesicles in the cytoplasm (blue). Scale bar = 25 μm. ***: *p* < 0.001 compared with the value at 0 h. Each point represents the mean ± standard deviation of eight independent measurements.

Finally, we investigated the use of the fluorogenic derivative **2c** in living cells by confocal microscopy. To this end, we used compounds **2c** and **3**, which were nontoxic to the cell line SH-SY 5Y after 24 h of treatment with a wide range of concentrations (Figure S9). As mentioned above, both derivatives produce a fluorescent signal in the violet region (excitation 405 nm, emission 420–470 nm) and can be captured by the confocal microscope (Figure 4c). However, when the Grob reaction takes place, two fragments are obtained that do not generate signal in this spectrum. Briefly, cells were treated with compounds **2c** and **3** (20 mM) for 24 h. At this time, cells were imaged by confocal microscopy, and satisfyingly, the loss of blue fluorescence in compound **2c** was observed as the result of the successful Grob fragmentation. On the other hand, the fluorescence intensity of compound **3** did not change over time (Figure S10). Finally, it is important to note that the substrates of Grob fragmentation undergo side reactions such as substitution, cyclization, or even elimination.^20^ However, we have not observed any potential compounds derived from these reactions with our derivatives and under the above experimental conditions.

In summary, we have designed and synthesized a new self-immolative linker based on the Grob fragmentation that allowed the controlled release of sulfonate-containing compounds such as a dansyl group under physiological conditions. We have also tuned conveniently the p*K*_a_ of the pushing group (amino group) using different substituents, leading to more efficient conversions at physiological pH and in some cases even at acidic pH, which is normally found in tumor environments. In addition, the Grob fragmentation takes place in living cells, demonstrating the potential bioorthogonal applicability of the reaction. On the basis of these promising results, research is currently underway to incorporate this type of linker into antibody–drug conjugates for the targeted delivery of cytotoxic drugs and fluorophores.

## Supporting Information Available

General information, synthesis, NMR studies, biology experimental details, confocal microscopy procedures, characterization data (PDF)
