# Supplementary material for: Bioorthogonal Self-Immolative Linker Based on Grob Fragmentation
Source: Org Lett. 2021 Oct 25;23(21):8580–4. doi: 10.1021/acs.orglett.1c03299 (PMC8576835; doi:10.1021/acs.orglett.1c03299)
Supplement: Supplementary file 1 — ol1c03299_si_001.pdf [file ol1c03299_si_001.pdf]

Bioorthogonal self-immolative linker based on Grob fragmentation

Xhenti Ferhati,[§,∇] Marina Salas-Cubero,[§,∇] Pablo Garrido ,[#] Josune García-Sanmartín,[#] Ana Guerreiro,[+] Alberto Avenoza,[§] Jesús H. Busto,[§] Jesús M. Peregrina,[§] Alfredo Martínez[#], Ester Jiménez-Moreno,[§,*] Gonçalo J.L. Bernardes,[+,‡,*] and Francisco Corzana[§,*]

[§] Departamento de Química, Centro de Investigación en Síntesis Química, Universidad de La Rioja, 26006 Logroño, La Rioja, Spain.

[#] Angiogenesis Group, Oncology Area, Center for Biomedical Research of La Rioja (CIBIR), 26006 Logroño, Spain.

[+] Instituto de Medicina Molecular Joao Lobo Antunes, Faculdade de Medicina de Universidad de Lisboa, 1649-028 Lisboa, Portugal.

[‡] Yusuf Hamied Department of Chemistry, University of Cambridge, Lensfield Road, Cambridge, CB2 1EW, United Kingdom.

Table of contents

1. General information 3
2. General procedures 3
3. Synthesis 4
4. NMR study of Grob fragmentation 10
5. Absorbance and emission spectra 17
6. Grob fragmentation studies in cell medium 17
7. Cell culture and toxicity assay 18
8. Confocal microscopy 19
9. Statistical analysis 20
10. NMR spectra 21
11. References 35

1. General information

Chemicals were purchased and used without further purification. Analytical thin layer chromatography (TLC) was performed on precoated aluminum backed plates with a 0.20 mm thickness of silica gel 60 with fluorescent indicator UV254 (Merck TLC Silica gel 60 F254). TLC plates were visualized with UV light and by staining with phosphomolybdic acid (PMA) solution (5 g of PMA in 100 mL of absolute ethanol) or sulfuric acid-ethanol solution (1:20). Column chromatography was performed on silica gel (230−400 mesh). ^{1}H, ^{13}C and ^{19}F NMR spectra were measured in the solvent stated at 500 MHz, 400 MHz or 300 MHz, 126, 100 or 75 MHz and 376 or 282 MHz spectrometers, respectively, with TMS as the internal standard. Chemical shifts are quoted in parts per million from residual solvent peak ($CDCl_3$: ^{1}H - 7.26 ppm and ^{13}C - 77.16 ppm) and coupling constants (*J*) given in Hertz. Multiplicities are quoted as singlet (s), broad singlet (brs), doublet (d), doublet of doublets (dd), triplet (t), or multiplet (m) or combinations thereof. Cq stands for quaternary carbon atom. Spectra were assigned using COSY and HSQC experiments. Magnitude-mode ge-2D COSY spectra were acquired with gradients by using the *cosygpqf* pulse program with a pulse width of 90º. Phase-sensitive ge-2D HSQC spectra were acquired by using z-filter and selection before t1 removing the decoupling during acquisition by using of the *invigpndph* pulse program with CNST2 (JHC)=145. All NMR chemical shifts (δ) were recorded in ppm and coupling constants (*J*) were reported in Hz. High resolution electrospray mass (ESI) spectra were recorded on a microTOF spectrometer; accurate mass measurements were achieved by using sodium formate as an external reference.

2. General procedures

General procedure A for the synthesis of secondary amines

To a solution of primary amine (5 equiv) in THF (0.2 M), the corresponding alkyl bromide (1 equiv) was added at 0 °C. The reaction was allowed to reach room temperature and stirred until complete starting material consumption. The solvent was removed under reduced pressure and the crude product used in the following step without further purification.

General procedure B for Boc protection of secondary amines

To a solution of the corresponding secondary amine in a CH_3CN:H_2O 2:1 mixture (0.15 M), a solution of $NaHCO_3$ (5%, 2.5 equiv) and Boc_2O (1.3 equiv) were added. The

reaction was stirred for 16 h at room temperature. Et_2O was then added, and the organic layer was washed with H_2O, dried over anhydrous Na_2SO_4, filtered and concentrated at reduced pressure. The residue was purified by column chromatography.

General procedures for Dansyl sulfonate formation

General procedure C: To a solution of the corresponding alcohol (1 equiv) in dry pyridine (0.2 M) under inert atmosphere at 0 °C, dansyl chloride (3 equiv) was added and the reaction was stirred overnight at room temperature. Afterwards, the crude mixture was concentrated to dryness under reduced pressure and purified by column chromatography.

General procedure D: To a solution of the corresponding alcohol (1 equiv) and DMAP (3 equiv) in dry CH_3CN (0.1 M) under N_2 atmosphere, a solution of dansyl chloride (3 equiv), in anhydrous CH_3CN (0.3 M) was added dropwise. The reaction mixture was then stirred for 16 h at room temperature until no starting alcohol was left (monitored by TLC). The reaction was concentrated to dryness under reduced pressure and the residue was purified by flash column chromatography.

General procedure E for Boc deprotection

N-Boc protected amines were treated with a 15% solution of TFA in CH_2Cl_2 (0.05 – 0.1 M). Reaction was monitored by TLC until no starting material was left. The reaction was concentrated *in vacuo* and co-evaporated with toluene to remove traces of TFA. The crude product was used in the following step without further purification.

3. Synthesis

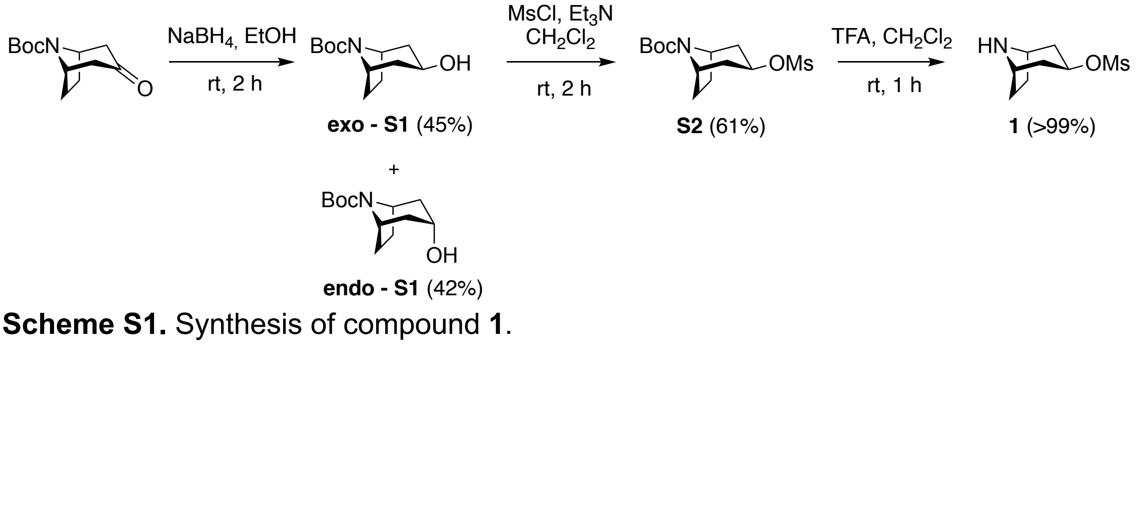

Scheme S1. Synthesis of compound **1**.

Synthesis of compound exo – S1: A solution of commercial *N*-Boc-nortropinone (920 mg, 4.44 mmol) in EtOH (44 mL) was cooled at 0 °C and treated with $NaBH_4$ (335 mg,

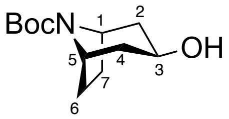

8.88 mmol). The reaction was allowed to warm at room temperature, and it was stirred for 2 h until TLC (AcOEt/hexanes 1:1) showed no starting material left. H_2O was then added (40 mL) and the mixture was stirred for 10 min at room temperature. After that, EtOH was evaporated under reduced pressure and the aqueous phase was extracted with AcOEt (3 x 50 mL). The combined organic phases were washed with brine (50 mL), dried over anhydrous Na_2SO_4, filtered, and concentrated *in vacuo*. The residue was purified by column chromatography (AcOEt/hexanes 1:2) to afford compounds **endo-S1** (425 mg, 1.87 mmol, 42%) and **exo-S1** (459 mg, 2.01 mmol, 45%) as pale yellow oils. Spectroscopic data for compound **exo-S1**: ^1H NMR (300 MHz, $CDCl_3$): δ (ppm) 4.17 (brs, 2H, H-1, H-5), 4.10 – 3.96 (m, 1H, H-3) 2.70 (m, 1H, OH) 1.92 – 1.84 (m, 4H, H-2, H-4, H-6, H-7), 1.59 – 1.46 (m, 4H, H-2, H-4, H-6, H-7) 1.42 (s, 9H, Boc). $^{13}C\{^1H\}$ NMR (75 MHz, $CDCl_3$): δ (ppm) 153.3 (C=O), 79.4 (Cq Boc), 63.6 (*C*H-3), 52.9 (2C, *C*H-1, *C*H-5), 40.3 (2C, *C*H_2-2, *C*H_2-4), 28.5 (3C, *C*H_3 Boc), 28.2 (2C, *C*H_2-6, *C*H_2-7). HRMS (ESI) m/z: $[M+Na]^+$ Calcd for $C_{12}H_{21}NO_3Na$ 250.1414; Found 250.1420. The spectroscopic data are in accordance to those reported in the literature.[1]

Synthesis of compound S2: To a solution of compound ***exo*-S1** (109 mg, 0.48 mmol)

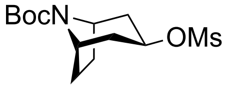

in anhydrous CH_2Cl_2 (2 mL) under inert atmosphere, MsCl (48 µL, 0.62 mmol) and NEt_3 (133 µL, 0.96 mmol) were added at 0 °C. The reaction was then allowed to warm at room temperature and stirred for 2 h. The reaction was then diluted with CH_2Cl_2 (50 mL) and washed with H_2O (2 x 20 mL). The organic phase was dried over anhydrous Na_2SO_4 and concentrated under reduced pressure. The residue was purified by column chromatography (AcOEt/hexanes 1:1) to afford compound **S2** (90 mg, 0.29 mmol, 61% yield) as a yellow oil. ^1H NMR (400 MHz, $CDCl_3$): δ (ppm) 5.02 (m, 1H, H-3), 4.23 (brs, 2H, H-1, H-5), 2.97 (s, 3H, OMs), 2.04 (brs, 2H, H-2, H-4), 1.96 (brs, 2H, H-6, H-7), 1.83 (brs, 2H, H-2, H-4), 1.65 – 1.63 (m, 2H, H-6, H-7), 1.44 (brs, 9H, Boc). $^{13}C\{^1H\}$ NMR (100 MHz, $CDCl_3$): δ (ppm) 153.1 (C=O), 79.9 (Cq Boc), 75.1 (*C*H-3), 52.7 (2C, *C*H-1, *C*H-5), 39.0 (*C*H_3 OMs), 37.7 (2C, *C*H_2-2, *C*H_2-4), 28.5 (3C, *C*H_3 Boc), 27.9 (2C, *C*H_2-6, *C*H_2-7). HRMS (ESI) m/z: $[M+Na]^+$ Calcd for $C_{13}H_{23}NO_5SNa$ 328.1189; Found 328.1190. The spectroscopic data are in accordance to those reported in the literature.[2]

Synthesis of compound 1: Following the general procedure E, compound **1** (40 mg, 0.13 mmol, 100%) was obtained as a transparent oil in quantitative yield from compound **S2** (50 mg, 0.13 mmol).

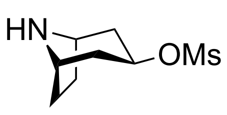

^{1}H NMR (300 MHz, CD_3OD): δ (ppm) 5.07 – 4.96 (m, 1H, H-3), 4.18 – 4.11 (m, 2H, H-1, H-5), 3.16 (s, 3H, OMs), 2.42 – 2.34 (m, 2H, H-2, H-4), 2.18 – 2.01 (m, 6H, H-2, H-4, H-6, H-7). $^{13}C\{^{1}H\}$ NMR (75 MHz, CD_3OD): δ (ppm) 73.1 (*C*H-3), 56.1 (2C, *C*H-1, *C*H-5), 38.3 (*C*H_3, OMs), 36.3 (2C, *C*H_2-2, *C*H_2-4), 26.8 (2C, *C*H_2-6, *C*H_2-7). HRMS (ESI) m/z: $[M+H]^+$ Calcd for $C_8H_{16}NO_3S$ 206.0845; Found 206.0852.

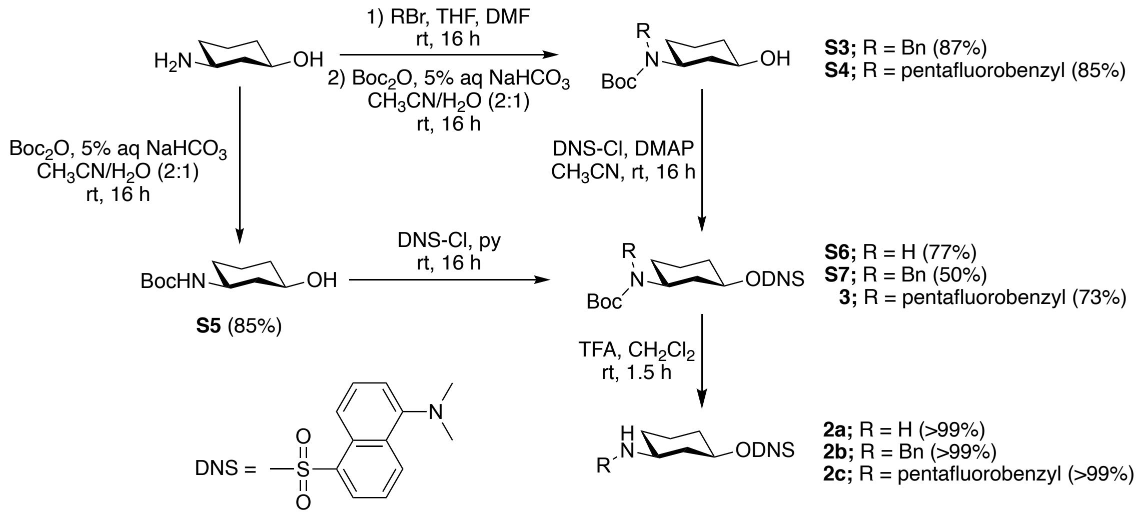

Scheme S2. Synthesis of compounds **2a**, **2b** and **2c**.

Synthesis of compound S3: Following general procedures A and B, compound **S3** (63 mg, 0.21 mmol, 87%) was obtained as a pale orange oil from (*1R,3S*)-3-aminocyclohexanol (138 mg, 1.2 mmol) and benzyl bromide (0.29 mL, 0.24 mmol), with addition of 20% DMF to achieve complete solubilization.

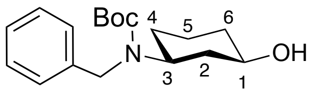

Then, the crude secondary amine was treated with Boc_2O (68 mg, 0.31 mmol) and an aqueous solution of $NaHCO_3$ (1 mL, 0.6 mmol) and the crude was purified by column chromatography (AcOEt/hexanes 1:2). ^{1}H NMR (500 MHz, $CDCl_3$): δ (ppm) 7.30 – 7.21 (m, 5H, H-Ar), 4.36 (brs, 2H, CH_2Ph), 4.07 (brs, H-3), 3.63 (brs, 2H, H-4, OH), 2.10 – 2.00 (m, 1 H), 1.91 (d, *J* = 11.6 Hz, 1 H), 1.76 – 1.73 (m, 1 H), 1.68 – 1.03 (m, 14H); $^{13}C\{^{1}H\}$ NMR (126 MHz, $CDCl_3$): δ (ppm) 155.8 (C=O), 140.3 (Cq arom.), 128.4, 126.8 (5C, *C*H arom.), 80.0 (Cq Boc), 70.1 (*C*H-1), 53.3 (*C*H-3), 46.8 (*C*H_2Ph), 40.6 (*C*H_2), 34.9 (*C*H_2), 29.9 (*C*H_2), 28.5 (3C, *C*H_3 *t*Bu), 22.3 (*C*H_2). MS (ESI) m/z: $[M+H]^+$ Calcd for $C_{18}H_{28}NO_3$ 306.21; Found 306.21. $[M+Na]^+$ Calcd for $C_{18}H_{27}NO_3Na$ 328.19; Found 328.19.

Synthesis of compound S4: Following general procedures A and B, compound **S4** (55 mg, 0.14 mmol, 85%) was obtained as a yellow oil from (*1R,3S*)-3-aminocyclohexanol (92 mg, 0.80 mmol) and 2,3,4,5,6-pentafluorobenzyl bromide (Pfb-Br, 42 mg, 0.16 mmol). Then, the crude secondary amine was treated with Boc_2O (45 mg, 0.21 mmol) and an aqueous solution of $NaHCO_3$ (1.68 mL, 0.4 mmol) and the crude was purified by column chromatography (AcOEt/hexanes 1:2). ^{1}H NMR (300 MHz, $CDCl_3$): δ (ppm) 4.56 (brs, 2H, CH_2 Pfb), 3.76 – 3.60 (m, 2H, H-1, H-3), 2.06 – 1.93 (m, 2H), 1.84 – 1.76 (m, 1H), 1.69 – 1.62 (m 2H), 1.46 (s, 9H, CH_3 Boc), 1.42 – 1.22 (m, 2H), 1.17 – 1.04 (m, 1H). ^{13}C{^{1}H} NMR (75 MHz, $CDCl_3$): δ (ppm) 155.0 (C=O), 146.6, 144.1, 138.8, 137.2, 136.3, 113.2, (6C, *C*-F arom.), 80.8 (Cq Boc), 70.0 (*C*H-1), 55.0 (*C*H-3), 40.0 (*C*H_2 Pfb), 37.0 (*C*H_2-2), 34.9 (*C*H_2), 28.4 (*C*H_2), 28.0 (3C, *C*H_3 Boc), 22.4 (*C*H_2-5). ^{19}F NMR (282 MHz, $CDCl_3$): δ (ppm) -142.8 (brs, 2F), -155.5 – -155.6 (m, 1F), -162.1 – -162.3 (m, 2F). HRMS (ESI) m/z: $[M+Na]^+$ Calcd for $C_{18}H_{22}F_5NO_3Na$ 418.1412; Found 418.1417.

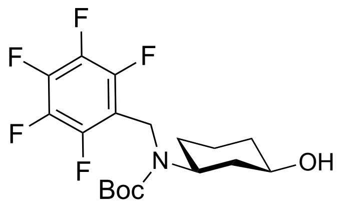

Synthesis of compound S5: Following general procedure B, compound **S5** (192 mg, 0.89 mmol, 85%) was obtained from (*1R,3S)*-3-aminocyclohexanol (120 mg, 1.05 mmol) as a pale-yellow oil by treatment with Boc_2O (360 mg, 1.65 mmol) and an aqueous solution of $NaHCO_3$ (5.3 mL, 3.17 mmol). The residue was purified by column chromatography (AcOEt/hexanes 1:1). ^{1}H NMR (400 MHz, $CDCl_3$): δ (ppm) 4.71 (brs, 1 H), 3.75 – 3.70 (m, 1H, H-1), 3.52 (brs, 1H, H-3), 2.19 – 2.16 (m, 1 H, H-2), 1.87 – 1.77 (m, 3H, H-4, H-5, H-6) 1.44 (s, 9H, CH_3 Boc) 1.37 – 1.06 (m, 4H, H-2, H-4, H-5, H-6). ^{13}C{^{1}H} NMR (100 MHz, $CDCl_3$): δ (ppm) 155.3 (C=O), 79.3 (Cq Boc), 69.1 (*C*H-1), 47.9 (*C*H-3), 41.8 (*C*H_2-2), 34.6, 32.4 (2C, *C*H_2-4, *C*H_2-6), 28.6 (3C, *C*H_3 Boc), 20.9 (*C*H_2-5). HRMS (ESI) m/z: $[M+H]^+$ Calcd for $C_{11}H_{22}NO_3$ 216.1594; Found 216.1596.

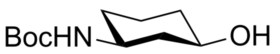

Synthesis of compound S6: Following general procedure C, compound **S6** (210 mg, 0.47 mmol, 77%) was obtained from compound **S5** (132 mg, 0.61 mmol) and dansyl chloride (DNS-Cl, 493.6 mg, 1.83 mmol) as a yellow oil. The residue was purified by column chromatography (AcOEt/hexanes 1:4). ^{1}H NMR (300 MHz, $CDCl_3$): δ (ppm) 8.68 (d, *J* = 8.6 Hz, 1H, H-DNS), 8.33 – 8.28 (m, 2H, DNS), 7.66 – 7.55 (m, 2H, DNS), 7.27-7.24 (m, 1H, DNS), 4.49 – 4.39 (m, 2H, H-1, NH), 3.43 (brs, 1H, H-3), 2.96 (s, 6H, 2CH_3 DNS) 2.14 – 2.07 (m, 1 H, H-2), 1.90 – 1.66 (m, 3H) 1.41 (s, 9H, CH_3 Boc) 1.39 – 1.03 (m, 4H). ^{13}C{^{1}H} NMR (75 MHz, $CDCl_3$): δ (ppm) 154.9 (C=O), 151.9, 132.6 (Cq), 131.6 (CH DNS), 130.2

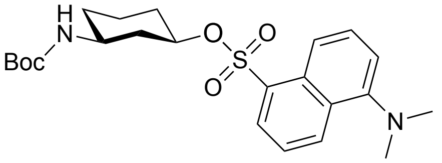

(Cq), 130.0 (CH DNS), 128.7 (CH DNS), 123.3 (CH DNS), 119.7, 115.6 (CH DNS), 80.0 (Cq Boc), 79.6 (CH-1), 47.5 (CH-3), 45.6 (2C, $2CH_3$ DNS), 38.9 (CH_2-2), 32.1, 31.8 (2C, CH_2-4, CH_2-6), 28.5 (3C, CH_3 Boc), 20.8 (CH_2-5). HRMS (ESI) m/z: $[M+H]^+$ Calcd for $C_{23}H_{33}N_2O_5S$ 449.2105; Found 449.2107.

Synthesis of compound S7: Following general procedure D, compound **S7** (29.6 mg, 0.06 mmol, 50%) was obtained from compound **S3** (44 mg, 0.11 mmol) and dansyl chloride (88.4 mg, 0.33 mmol) as a pale orange oil. The residue was purified by column chromatography (AcOEt/hexanes 1:4). ^{1}H NMR (400 MHz, $CDCl_3$): δ (ppm) 8.61 (d, *J* = 8.3 Hz, 1H, H DNS), 8.26 (d, *J* = 7.2 Hz, 1H, H DNS), 8.19 (d, *J* = 8.6 Hz, 1H, H DNS), 7.56 – 7.51 (m, 2H, H DNS), 7.26 – 7.19 (m, 4H, 1H DNS, 3H Ph), 7.10 –7.09 (m, 2H, Ph), 4.34 – 4.26 (m, 3H, C*H₂*Ph, H-1), 3.91 – 3.44 (m, 1H, H-3), 2.90 (s, 6H, $2CH_3$ DNS), 1.99 – 1.96 (m, 1H), 1.84 – 1.81 (m, 1H), 1.66 – 1.54 (m, 3H), 1.33 – 1.12 (m, 12H). $^{13}C\{^1H\}$ NMR (100 MHz, $CDCl_3$): δ (ppm) 155.5 (C=O), 151.7 (Cq), 139.7 (Cq), 132.6 (Cq), 131.5 (CH DNS), 130.1 (Cq), 129.9 (CH DNS), 128.6 (CH DNS), 128.4 (2C, Ph), 126.9, 126.5 (4C, Cq, Ph) 123.3 (CH DNS), 119.8 (CH DNS), 115.6 (CH DNS), 80.6 (Cq Boc), 80.2 (CH-1), 53.6 (CH-3), 46.9 (*C*H_2-Ph), 45.6 (2C, $2CH_3$ DNS), 37.2 (CH_2), 31.9 (CH_2), 29.5 (CH_2), 28.4 (3C, CH_3 Boc), 21.8 (CH_2). HRMS (ESI) m/z: $[M+H]^+$ Calcd for $C_{30}H_{39}N_2O_5S$ 539.2574; Found 539.2575.

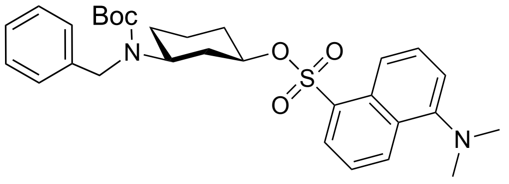

Synthesis of compound 3: Following general procedure D, compound **3** (46 mg, 0.073 mmol, 73%) was obtained from compound **S4** (40 mg, 0.10 mmol), dansyl chloride (80.9 mg, 0.3 mmol) and DMAP (36.7 mg, 0.3 mmol) as a pale-yellow oil. The residue was purified by column chromatography (AcOEt/hexanes 1:4). ^{1}H NMR (400 MHz, $CDCl_3$): δ (ppm) 8.61 (d, *J* = 8.4 Hz, 1H, H DNS), 8.26 (d, *J* = 7.3 Hz, 1H, H DNS), 8.20 (d, *J* = 8.6 Hz, 1H, H DNS), 7.61 – 7.49 (m, 2H, H DNS), 7.21 (d, *J* = 7.5 Hz, 1H, H DNS), 4.43 – 4.30 (m, 3H, CH_2 Pfb, H-1), 3.52 (brs, 1H, H-3), 2.90 (s, 6H, $2CH_3$ DNS), 1.87 (brs, 2H), 1.71 – 1.68 (m, 2H), 1.57 – 1.54 (m, 1H), 1.35 (s, 9H, Boc), 1.33 – 1.10 (m, 3H). $^{13}C\{^1H\}$ NMR (100 MHz, $CDCl_3$): δ (ppm) 154.7 (Cq, C=O), 151.7 (Cq DNS), 145.9 (d, *J* = 145.3 Hz, 2C, C-F Pfb), 140.5 (d, *J* = 140.5, C-F Pfb), 137.2 (m, 2C, C-F Pfb), 132.7 (Cq DNS), 131.5 (CH DNS), 130.1 (CH DNS), 130.0 (Cq DNS), 129.9 (Cq DNS), 128.5 (CH DNS), 123.3 (CH DNS), 119.8 (CH DNS), 115.7 (CH DNS), 112.8 (m, C-F Pfb), 81.0 (Cq Boc), 80.4 (CH-1), 54.9 (CH-3), 45.6 (2C, $2CH_3$ DNS), 37.3 (CH_2 Pfb),

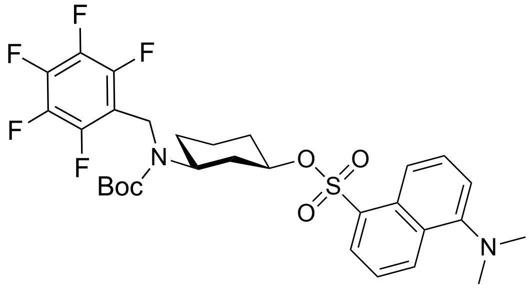

36.8 (CH_2), 31.9 (CH_2), 29.8 (CH_2), 28.9 (CH_2), 28.3 (3C, CH_3 Boc), 21.9 (CH_2). ^{19}F NMR (376 MHz, $CDCl_3$): δ (ppm) -146.6 (s, 2F), -159.1 (s, 1F), -165.9 (td, *J* = 21.6, 7.4 Hz). HRMS (ESI) m/z: $[M+H]^+$ Calcd for $C_{30}H_{34}F_5N_2O_5S$ 629.2106; Found 629.2103.

Synthesis of compound 2a: Following the general procedure E, compound **2a** (9 mg, 0.024 mmol, 100%) was obtained from compound **S6** (11 mg, 0.024 mmol) as a pale-yellow oil. ^{1}H NMR (300 MHz, CD_3OD): δ (ppm) 8.68 (d, *J* = 8.6 Hz, 1H, H DNS), 8.34 (dd, *J* = 7.3, 1.3 Hz, 1H, H DNS), 8.27 (d, *J* = 8.7 Hz, 1H, H DNS), 7.69 – 7.66 (m, 2H, H DNS), 7.39 (d, *J* = 7.7 Hz, 1H, H DNS), 4.56 – 4.38 (m, 1H, H-1), 3.19 – 3.06 (m, 1H, H-3), 2.94 (s, 6H, 2CH_3 DNS), 2.23 (m, 1H, H-2), 1.95 – 1.73 (m, 3H, H-4, H-5, H-6), 1.54 (q, *J* = 11.6 Hz, 1H, H-2), 1.40 – 1.23 (m, 3H, H-4, H-5, H-6). ^{13}C{^{1}H} NMR (75 MHz, CD_3OD): δ (ppm) 151.0 (Cq DNS), 133.9 (Cq DNS), 131.9 (CH DNS), 131.6 (CH DNS), 131.0 (Cq DNS), 130.5 (Cq DNS), 129.6 (CH DNS), 125.1 (CH DNS), 122.0 (CH DNS), 117.6 (CH DNS), 79.9 (CH-1), 47.4 (CH-3), 46.1 (2C, 2CH_3 DNS), 37.7 (CH_2-2), 32.3 (CH_2-6), 30.1 (CH_2-4), 21.5 (CH_2-5). HRMS (ESI) m/z: $[M+H]^+$ Calcd for $C_{18}H_{25}N_2O_3S$ 349.1580; Found 349.1586.

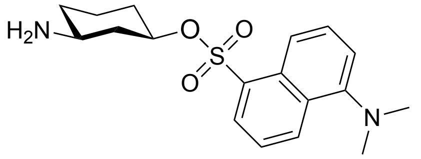

Synthesis of compound 2b: Following general procedure E, compound **2b** (12 mg, 0.028 mmol, 100%) was obtained from compound **S7** (15 mg, 0.028 mmol) as a yellow oil. ^{1}H NMR (400 MHz, CD_3OD): δ (ppm) 8.65 (d, *J* = 8.6 Hz, 1H, H DNS), 8.32 (dd, *J* = 7.3, 1.0 Hz, 1H, H DNS), 8.28 (d, *J* = 8.7 Hz, 1H, H DNS), 7.73 – 7.55 (m, 2H, H DNS), 7.49 – 7.28 (m, 6H, Ph, H DNS), 4.43 (m, 1H, H-1), 4.15 (d, *J* = 13.0 Hz, 1H, C*H₂*Ph), 4.08 (d, *J* = 13.0 Hz, 1H, C*H₂*Ph), 3.20 – 3.04 (m, 1H, H-3), 2.96 (s, 6H, CH_3 DNS), 2.40 – 2.31 (m, 1H, H-2), 2.05 (m, 1H, H-4), 1.85 – 1.69 (m, 2H, H-5, H-6), 1.57 (q, *J* = 11.7 Hz, 1H, H-2), 1.44 – 1.15 (m, 3H, H-4, H-5, H-6). ^{13}C{^{1}H} NMR (100 MHz, CD_3OD): δ (ppm) 151.2 (Cq DNS), 133.8 (Cq DNS), 132.4 (Cq Ph), 132.0 (CH DNS), 131.6 (CH DNS , 131.0 (Cq DNS), 130.8 (2CH Ph), 130.7 (2CH Ph), 130.5 (Cq DNS), 130.3 (CH Ph), 129.7 (CH DNS), 125.0 (CH DNS), 121.9 (CH DNS), 117.6 (CH DNS), 79.9 (CH-1), 55.8 (CH-3), 48.0 (*C*H_2Ph), 46.1 (2C, 2CH_3 DNS), 36.3 (CH_2-2), 32.4 (CH_2-6), 28.4 (CH_2-4), 21.5 (CH_2-5). HRMS (ESI) m/z: $[M+H]^+$ Calcd for $C_{25}H_{31}N_2O_3S$ 439.2050; Found 439.2050.

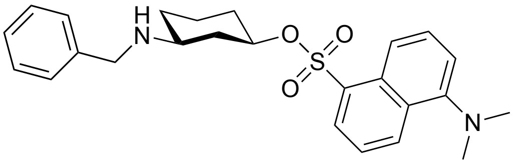

Synthesis of compound 2c: Following general procedure E, compound **2c** (13 mg, 0.024 mmol) was obtained from compound **3** (15 mg, 0.024 mmol) as a pale-yellow oil.

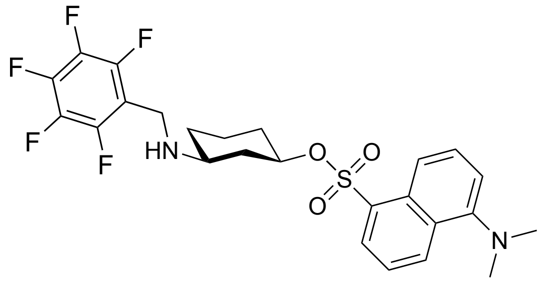

^{1}H NMR (400 MHz, CD_3OD): δ (ppm) 8.67 (d, *J* = 8.6 Hz, 1H, H DNS), 8.36 (t, *J* = 8.6 Hz, 2H, H DNS), 7.75 – 7.68 (m, 2H, H DNS), 7.51 (d, *J* = 7.6 Hz, 1H), 4.57 – 4.47 (m, 1H, H-1), 4.35 (d, *J* = 14.0, 1H, CH_2 Pfb), 4.27 (d, *J* = 14.0, 1H, CH_2 Pfb), 3.31-3.24 (m, 1H, H-3), 3.02 (s, 6H, $2CH_3$ DNS), 2.44 – 2.39 (m, 1H, H-2), 2.15 – 2.12 (m, 1H, H-4), 1.89 – 1.82 (m, 2H, H-5, H-6), 1.64 (q, *J* = 11.5 Hz, 1H, H-2), 1.49 – 1.22 (m, 3H, H-6, H-4, H-5). $^{13}C\{^1H\}$ NMR (100 MHz, CD_3OD): δ (ppm) 151.1 (Cq DNS), 147.2 (m, *J* = 246.1, 2C, C-F Pfb), 143.9 (d, *J* = 256.0, C-F Pfb), 139.1 (d, *J* = 149.6 Hz, 2C, C-F Pfb), 133.9 (Cq DNS), 132.0 (CH DNS), 131.6 (CH DNS), 131.0 (Cq DNS), 130.5 (Cq DNS), 129.7 (CH DNS), 125.1 (CH DNS), 122.0 (CH DNS), 117.7 (CH DNS), 106.9 (m, Cq Pfb), 79.7 (CH-1), 56.7 (CH-3), 46.1 (2C, $2CH_3$ DNS), 36.3 (*C*H_2 Pfb), 36.2 (CH_2-2), 32.4 (CH_2-6), 28.2 (CH_2-4), 21.4 (CH_2-5). ^{19}F NMR (376 MHz, CD_3OD): δ (ppm) -77.45 (CF_3 TFA), -141.75 – -141.83 (m, 2F), -153.46 – -153.58 (m, 1F), -163.57 – -163.72 (m, 2F). HRMS (ESI) m/z: $[M+H]^+$ Calcd for $C_{25}H_{26}F_5N_2O_3S$ 529.1579; Found 529.1573.

4. NMR study of Grob fragmentation

Compound **1** was dissolved in a mixture 1:1 of CH_3OH/buffer (PBS, NaPi 0.1M pH 8.0, NaPi 0.1 M pH 9.3) at a final concentration of 20 mM. After 18 h, the solution was concentrated under reduced pressure and a ^{1}H NMR spectrum in CD_3OD was recorded.

Compounds **2a**, **2b**, **2c** and **3** were dissolved in a mixture 1:1 of CD_3CN/deuterated buffer (PBS, NaPi 0.1 M pH 6.0, NaPi 0.1 M pH 8.0) at a final concentration of 5 mM. NMR tubes were incubated at 37 °C and various ^{1}H NMR spectra were recorded at different times.

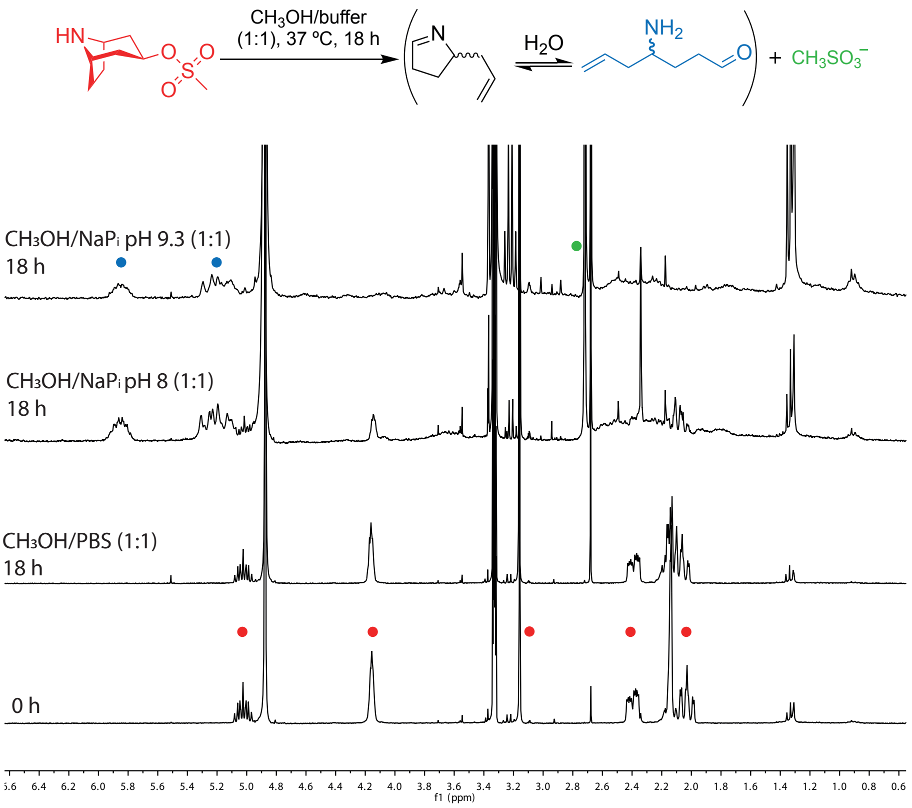

Figure S1. ^{1}H NMR release studies of **1**. The reaction was performed at 20 mM of **1** in a mixture 1:1 of CH_3OH/buffer (PBS, NaPi 0.1 M pH 8.0, NaPi 0.1 M pH 9.3).

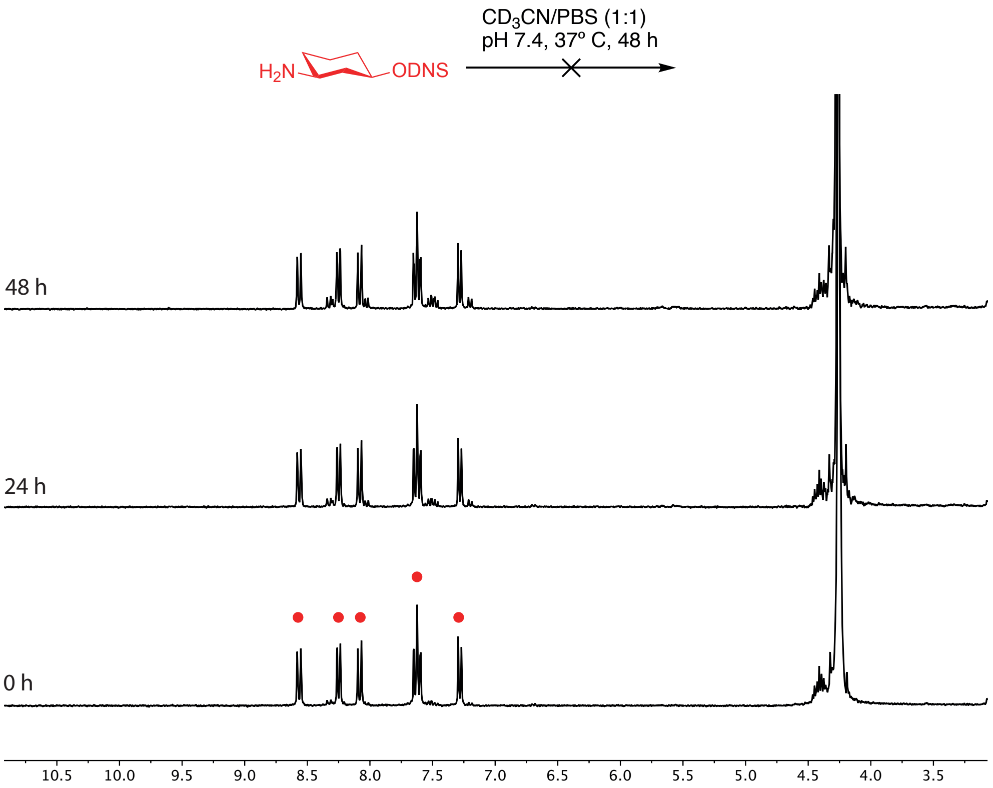

Figure S2. ^{1}H NMR release studies of **2a**. The reaction was performed at 5 mM of **2a** in CD_3CN/PBS pH 7.4 (1:1) at 37 ºC.

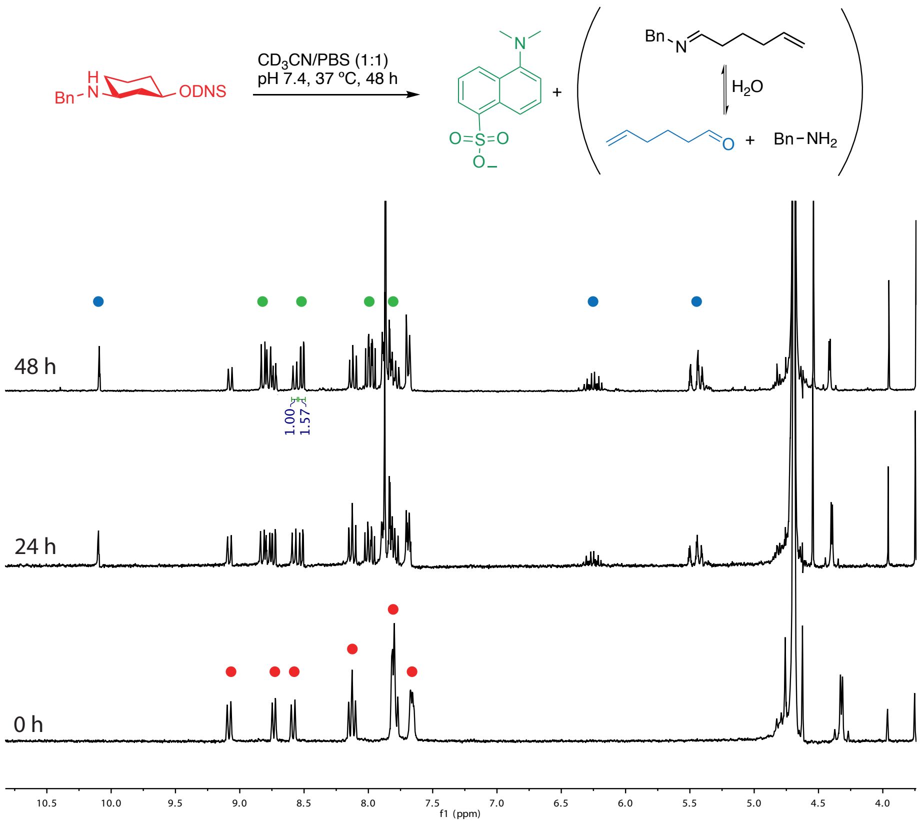

Figure S3. ^{1}H NMR release studies of **2b**. The reaction was performed at 5 mM of **2b** in CD_3CN/PBS pH 7.4 (1:1) at 37 ºC. Integration of selected peaks at 48 h is shown.

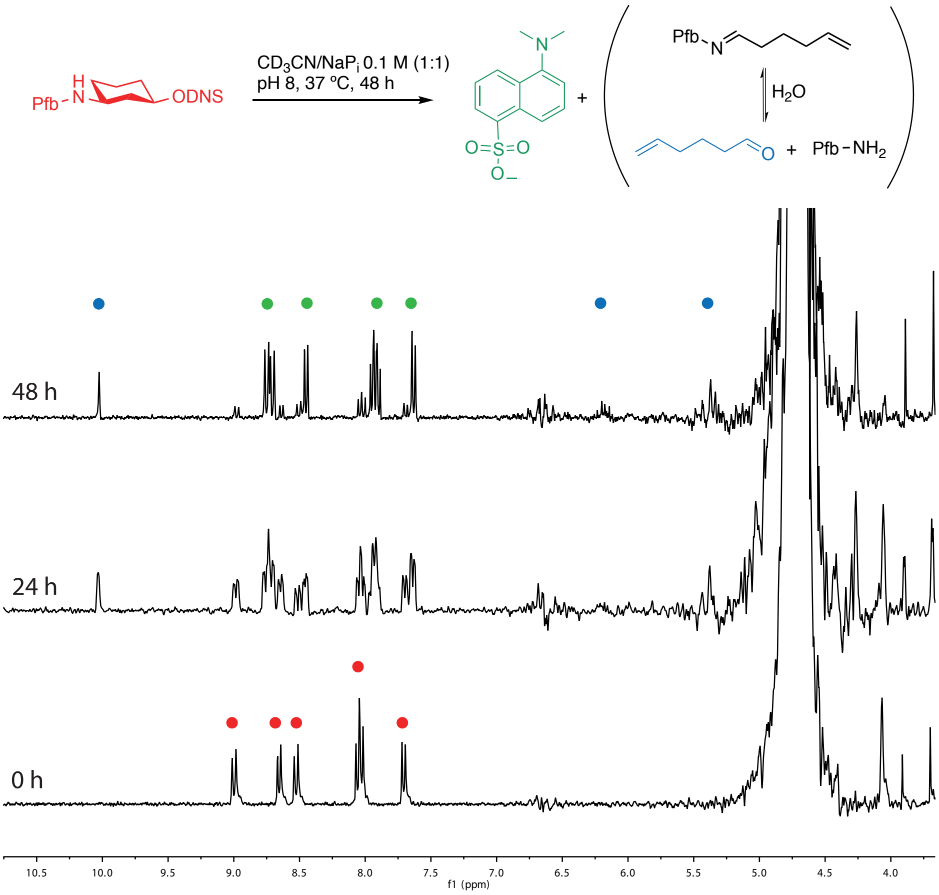

Figure S4. ^{1}H NMR release studies of **2c**. The reaction was performed at 5 mM of **2c** in CD_3CN/NaP_i 0.1 M pH 8.0 (1:1) at 37 ºC. Integration at 48 h was not possible due to the low signal to noise ratio.

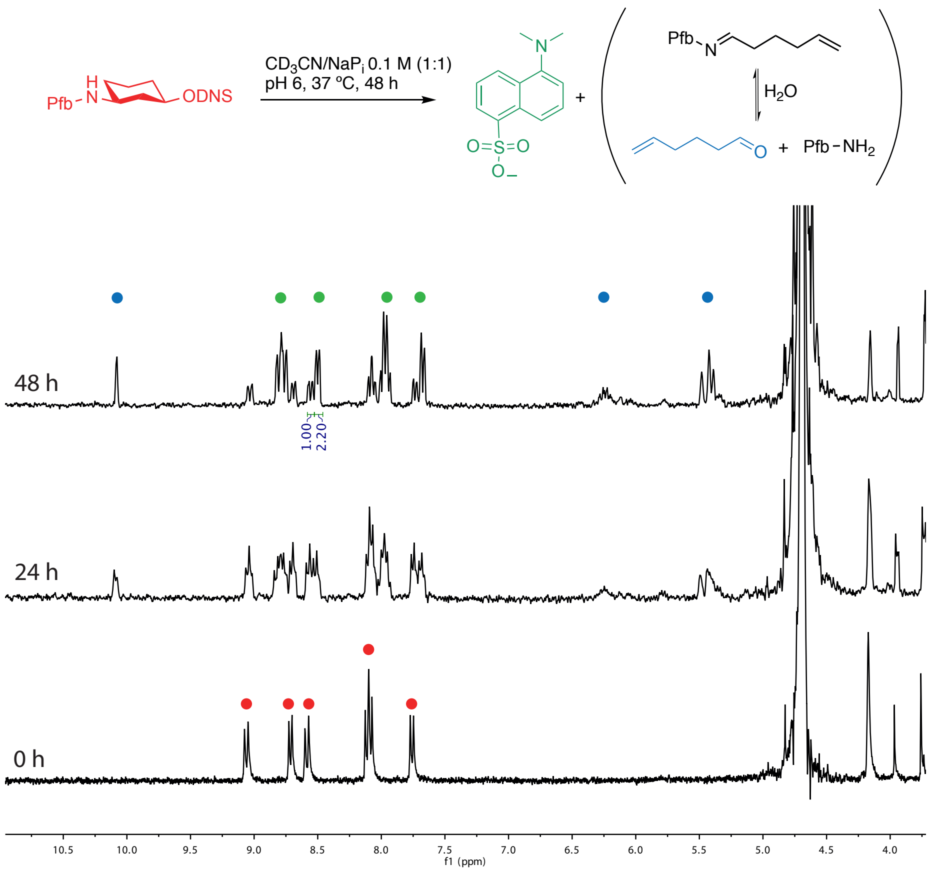

Figure S5. ^{1}H NMR release studies of **2c**. The reaction was performed at 5 mM of **2c** in CD_3CN/NaP_i 0.1 M pH 6.0 (1:1) at 37ºC. Integration of selected peaks at 48 h is shown.

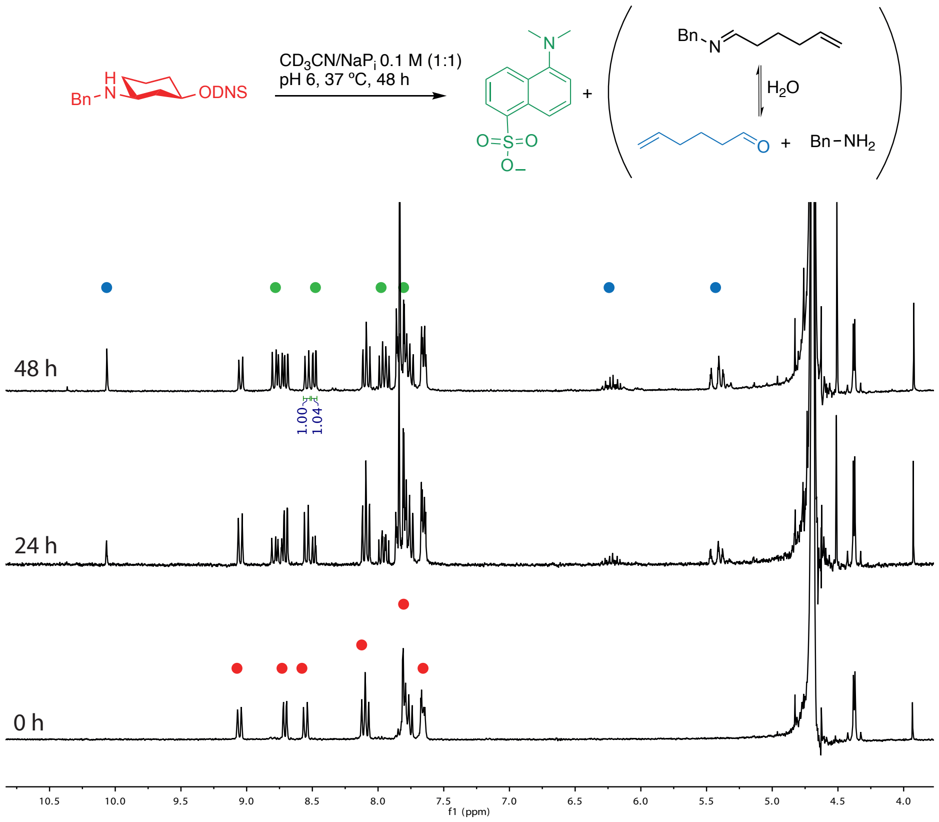

Figure S6. ^{1}H NMR release studies of **2b**. The reaction was performed at 5 mM of **2b** in CD_3CN/NaP_i 0.1 M pH 6.0 (1:1) at 37 ºC. Integration of selected peaks at 48 h is shown.

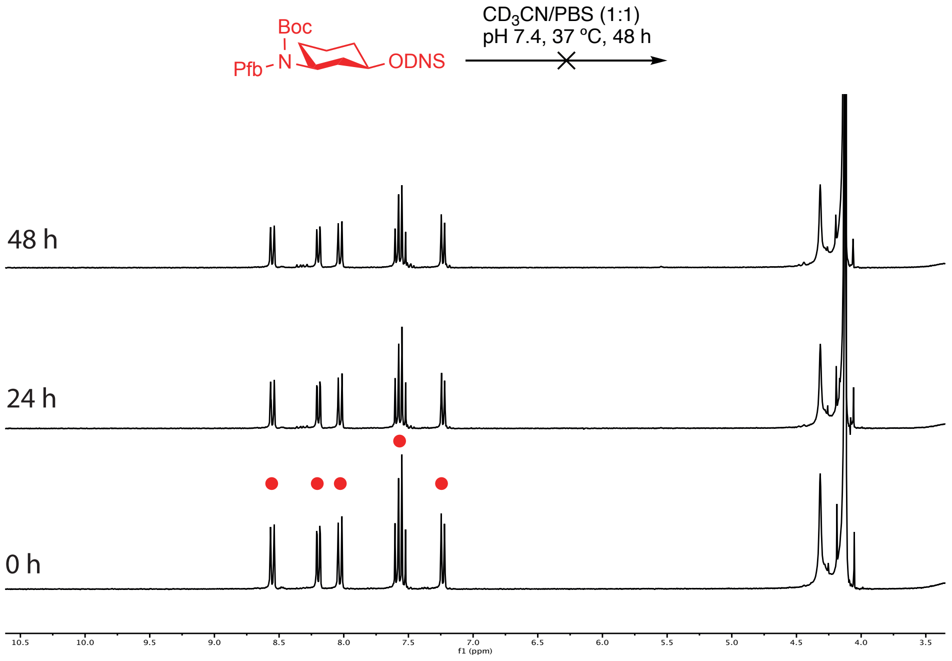

Figure S7. ^{1}H NMR release studies of **3**. The reaction was performed at 5 mM of **3** in CD3CN/PBS pH 7.4 (1:1) at 37 °C.

5. Absorbance and emission spectra

Dansyl-OH and compounds **2c** and **3** (5 mg) were dissolved in methanol (3 mL). Absorbance and emission spectra were measured in an Edinburgh FLS 1000. See Figure 4a in the main text.

6. Grob fragmentation studies in cell medium

Compounds **2c** and **3** were added to cell culture medium at different concentrations (10, 20, or 30 μM) and pH (7.5 or 6.0) and were incubated for different time periods (0, 4, 24, 28, 48 h) at 37 °C. Medium aliquots were transferred to 96-well plates and analyzed in a microplate reader (Synergy 4, BioTek) at 380 nm (excitation) and 528 nm (emission).

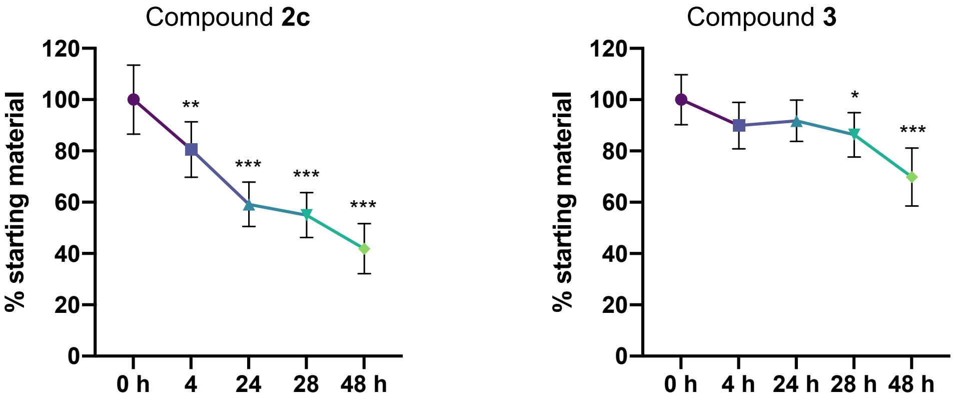

Figure S8. Evolution of Grob fragmentation for compounds **2c** and **3** (20 μM) in cell medium (pH 6.0). *: $p < 0.05$; **: $p < 0.01$; ***: $p < 0.001$ compared with the value at 0 h. Each point represents the mean ± standard deviation of 8 independent measurements.

7. Cell culture and toxicity assay

Human neuroblastoma SH-SY 5Y cell line was obtained from the ATCC (American Type Culture Collection). Cells were grown in DMEM-F12 medium (Hyclone) with 10% fetal bovine serum (Gibco), 1% Penicillin/Streptomycin (Gibco), and maintained at 37 °C, 5% CO_2. Cell culture medium was changed three times a week. The cell line was authenticated by STR profiling (IDEXX BioAnalytics).

Cell survival was analyzed using the CellTiter 96 Aqueous One Solution Cell Proliferation Assay (Promega), following manufacturer's instructions. Cells were seeded in 96 well plates at a density of 2 x 10^4 cells per well, allowed to attach for 24 h, and exposed to different concentrations (0.39 to 100 μM) of the compounds in DMSO for a further 24 h. DMSO was used as a control at the same dilutions. The MTS reagent was added (20 μL/well) and incubated for 4 h. Absorbance was examined at 490 nm using a microplate reader (POLARstar Omega, BMG Labtech).

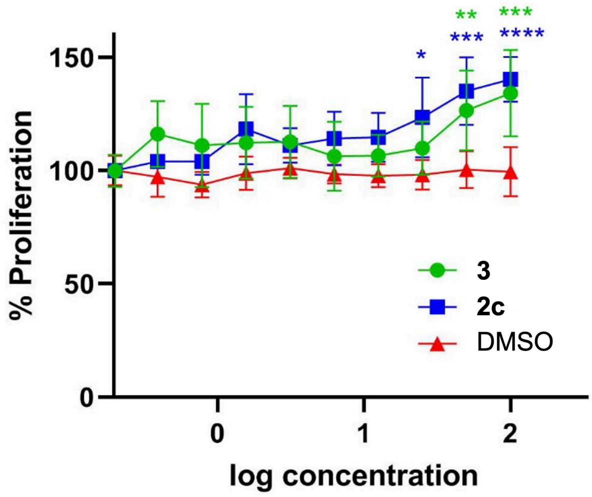

Figure S9. SH-SY 5Y cells were incubated in the presence of compounds **3** and **2c** (and DMSO as a control) for 24 h. They were then subjected to an MTS proliferation/survival assay. No toxicity was found at any concentration tested. In fact, the highest concentrations of compounds **3** and **2c** resulted in a significant increase in cell proliferation. Bars represent the mean ± standard deviation of 8 independent measurements. *: $p < 0.05$; **: $p < 0.01$; ***: $p < 0.001$; ****: $p < 0.0001$ compared with the control.

8. Confocal microscopy

SH-SY 5Y cells were seeded in glass-bottom 35 mm dishes (MatTek) and allowed to attach for 24 h. Then, they were exposed to the experimental compounds (at 20 μM). Cell membranes were labeled with 1:1.000 CellMask Orange (C10045, Thermofisher). After 1 h, the first images were taken with a confocal microscope (TCS SP5, Leica) in two channels: violet (excitation 405 nm, emission 420-470 nm) and yellow/orange (excitation 543 nm, emission 555-620 nm). Further images were taken at 24 h. The images were processed using the free software *ImageJ* and two parameters were measured as shown in Figure S10. The changes of both parameters are not statistically significant for compound **3**, whereas a significant ($p < 0.01$) time-dependent decrease is observed for derivative **2c**.

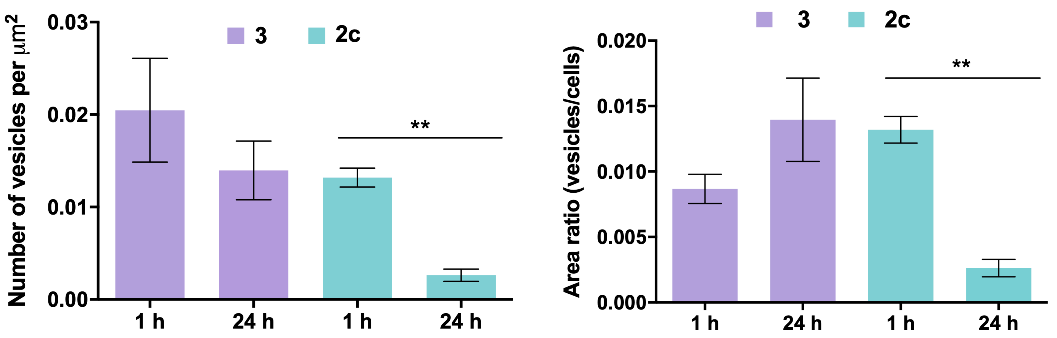

Figure S10. Left panel: Number of compound particles/vesicles in each area of cell surface (1.0 μm^2). Right panel: area occupied by the compounds divided by the area occupied by the cells. Each bar represents the mean ± standard deviation of 6 independent measurements. **: $p < 0.01$.

9. Statistical analysis

All datasets were tested for normalcy and homoscedasticity with the Shapiro-Wilk´s and Levene´s tests, respectively. Time evolution and cell survival were tested with 1-way ANOVA, followed by Dunnet´s post-hoc test. *p* values lower than 0.05 were considered statistically significant. All analyses were performed with the Prism 8.3.0 software package.

10. NMR spectra

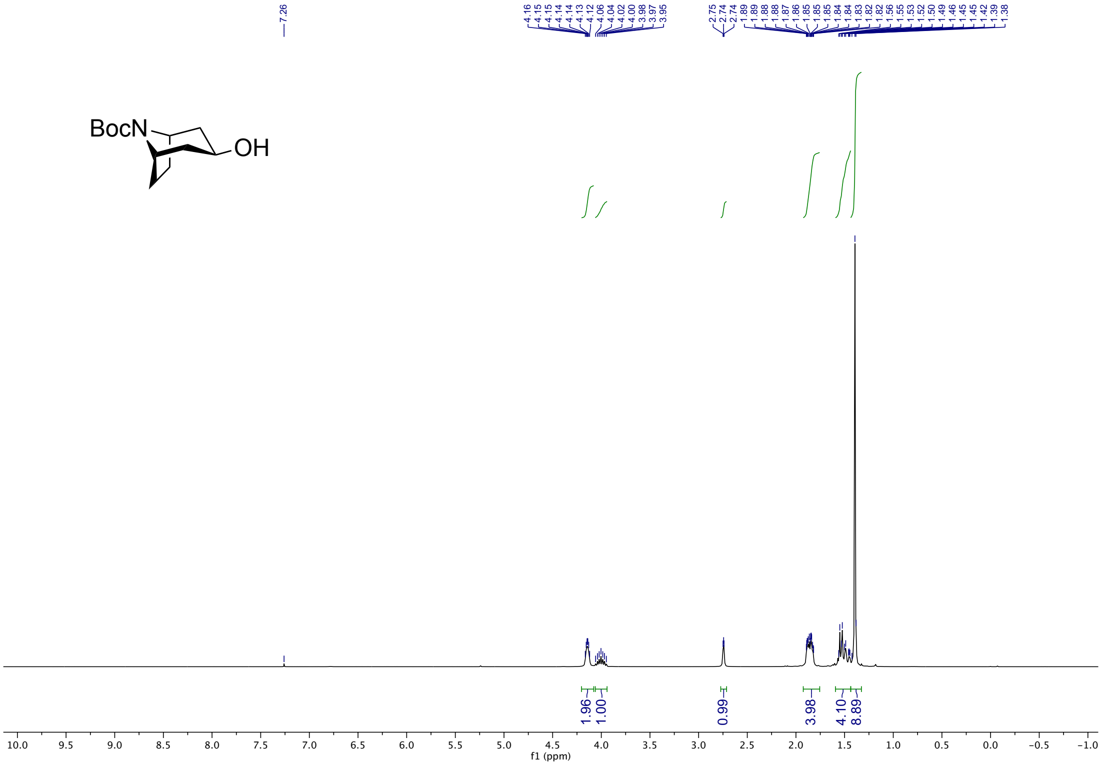

Figure S11. ^{1}H NMR (300 MHz, $CDCl_3$) of compound **S1**

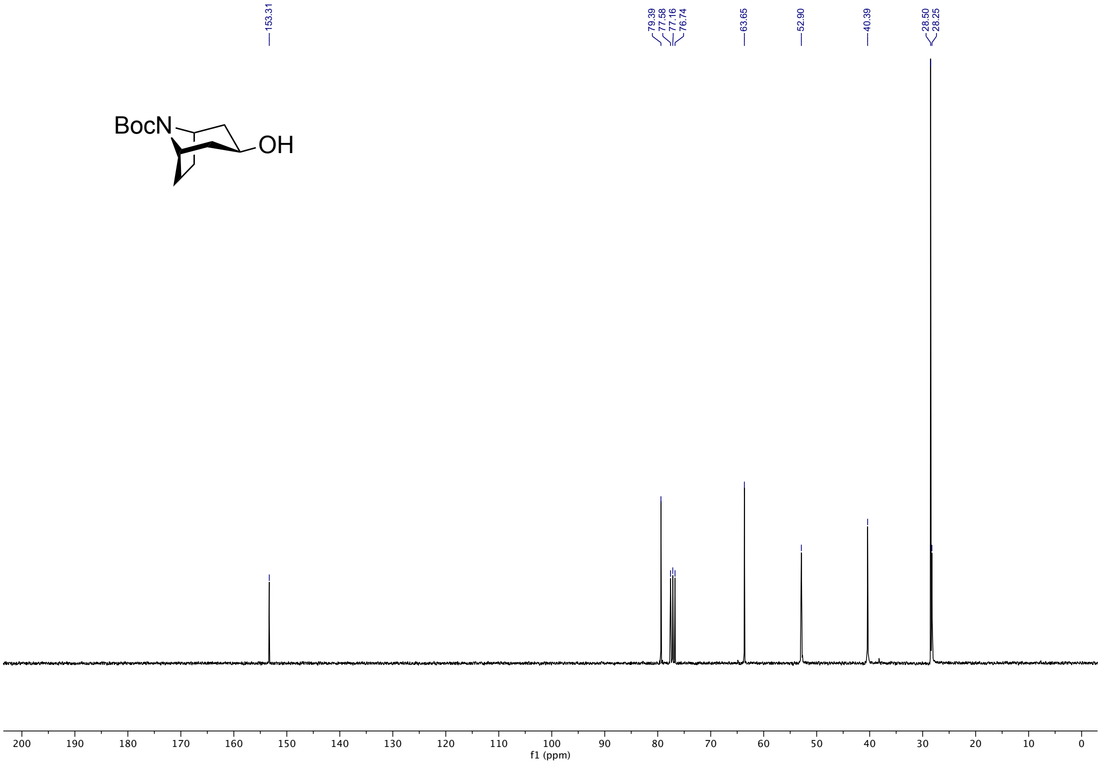

Figure S12. ^{13}C{^{1}H} NMR (75 MHz, $CDCl_3$) of compound **S1**

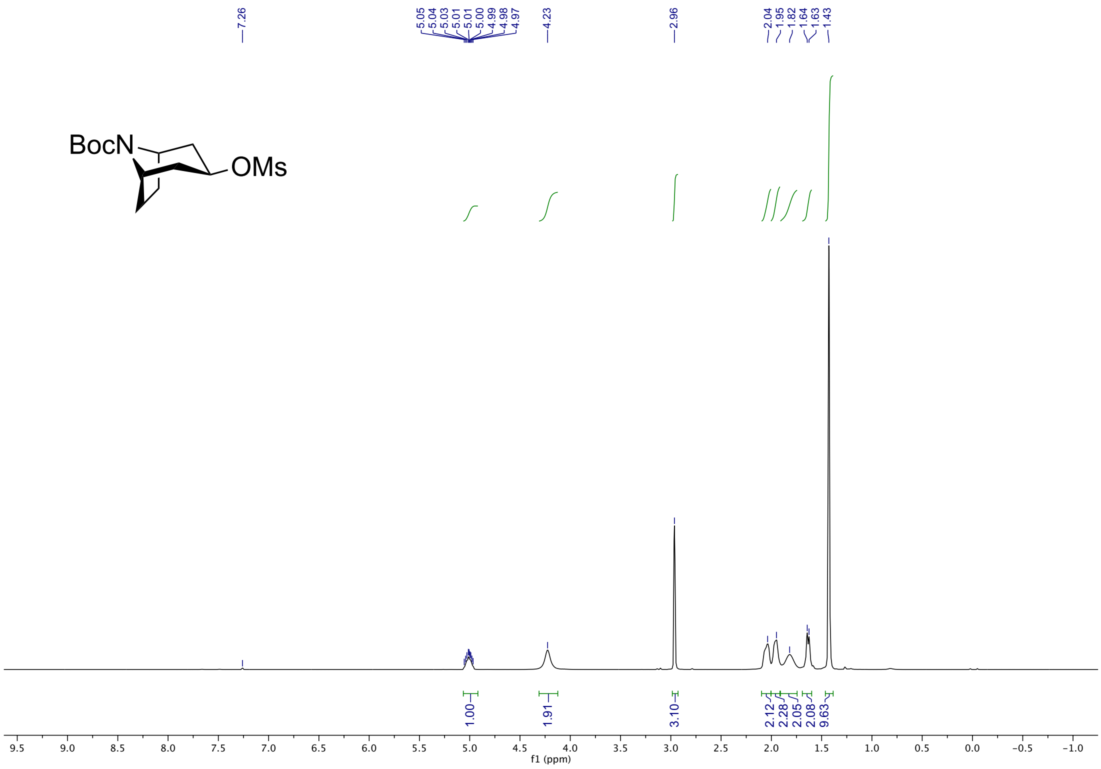

Figure S13. ^{1}H NMR (400 MHz, $CDCl_3$) of compound **S2**

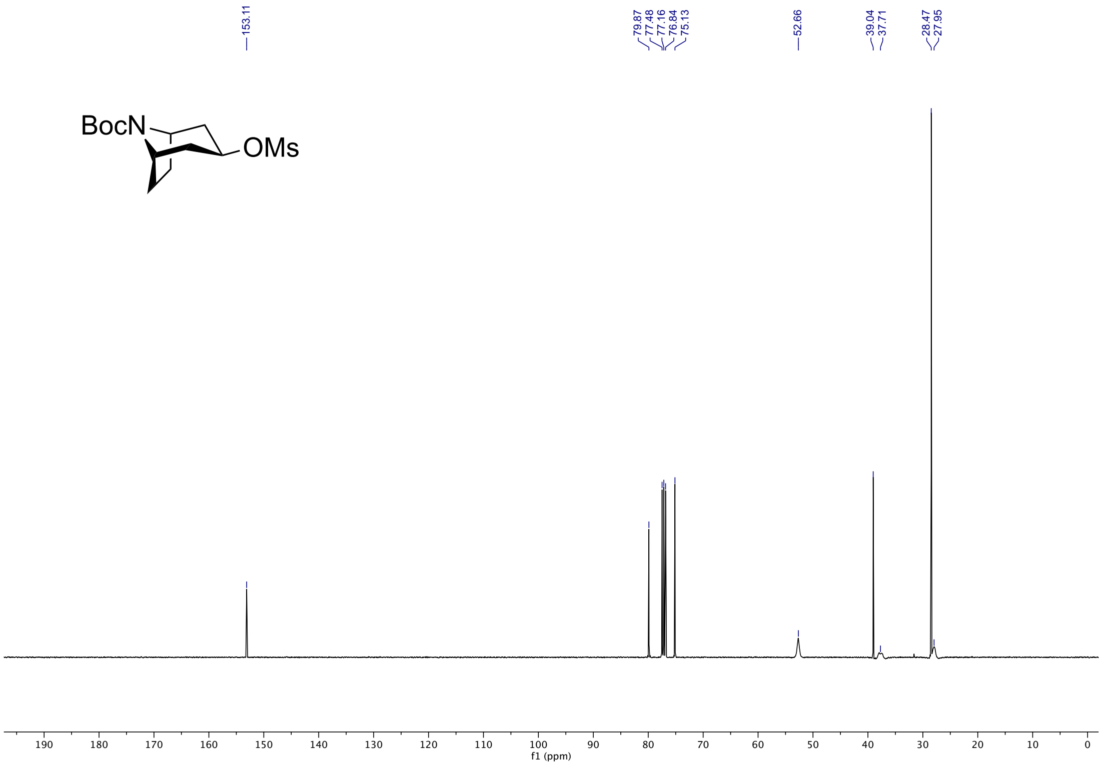

Figure S14. $^{13}C\{^{1}H\}$ NMR (100 MHz, $CDCl_3$) of compound **S2**

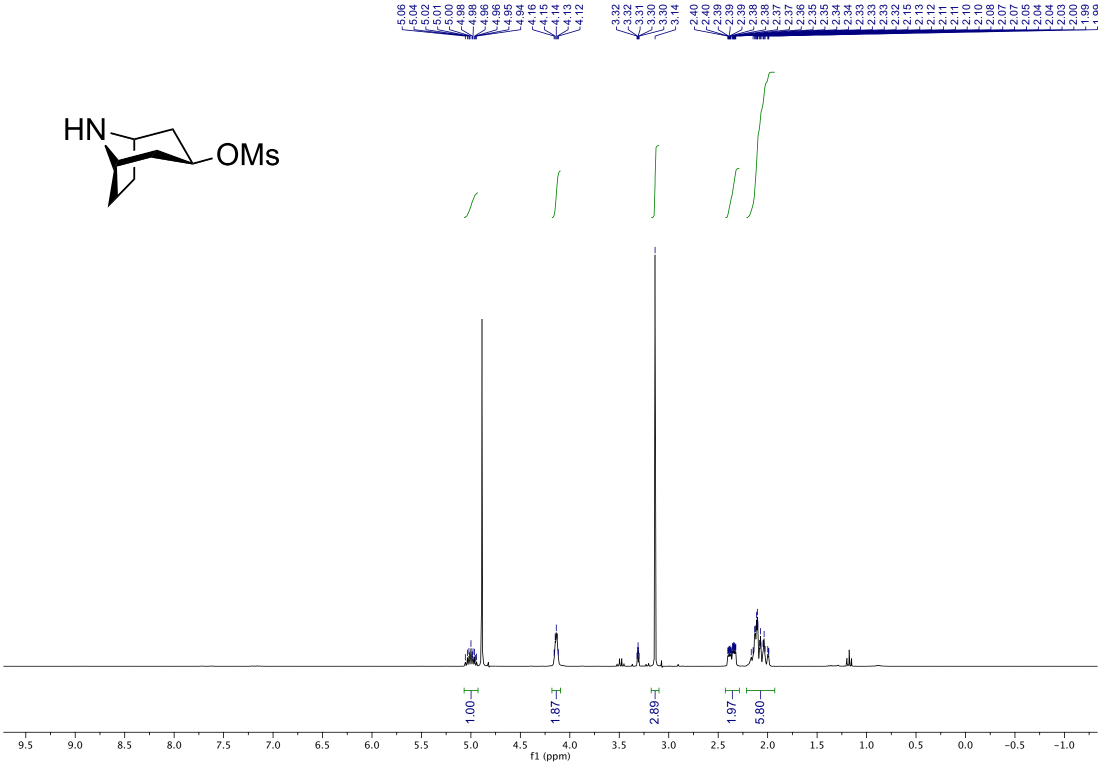

Figure S15. ^{1}H NMR (300 MHz, CD_3OD) of compound **1**

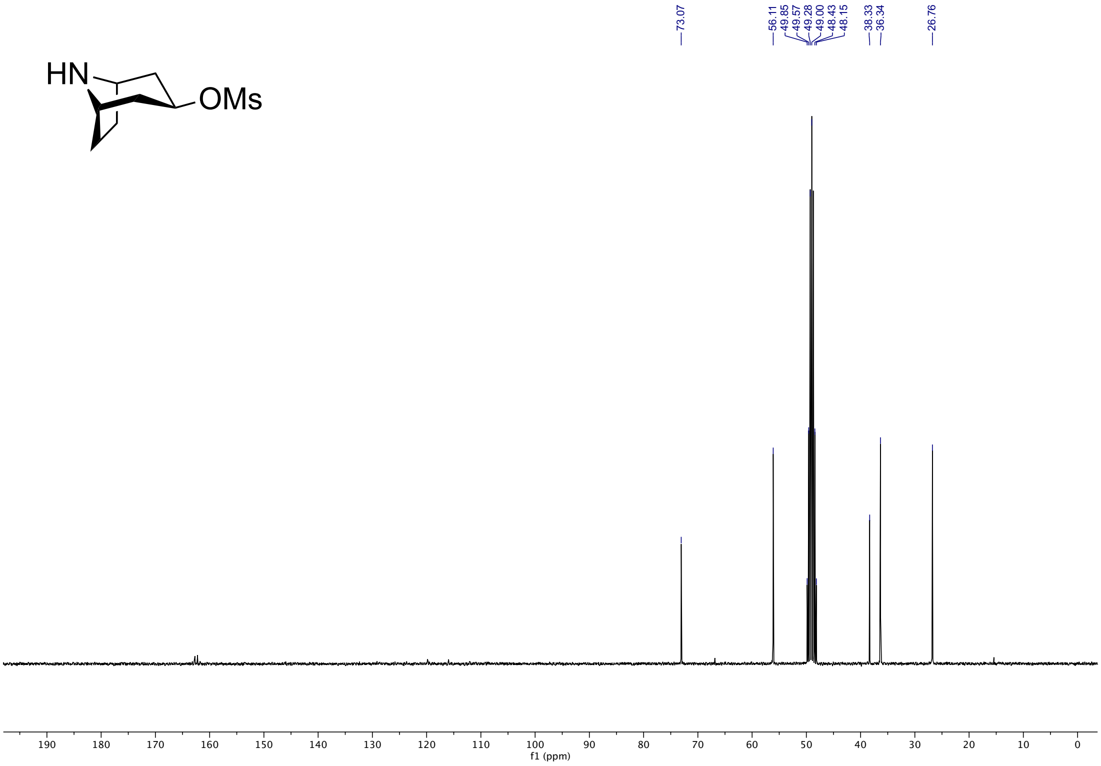

Figure S16. $^{13}C\{^{1}H\}$ NMR (75 MHz, CD_3OD) of compound **1**

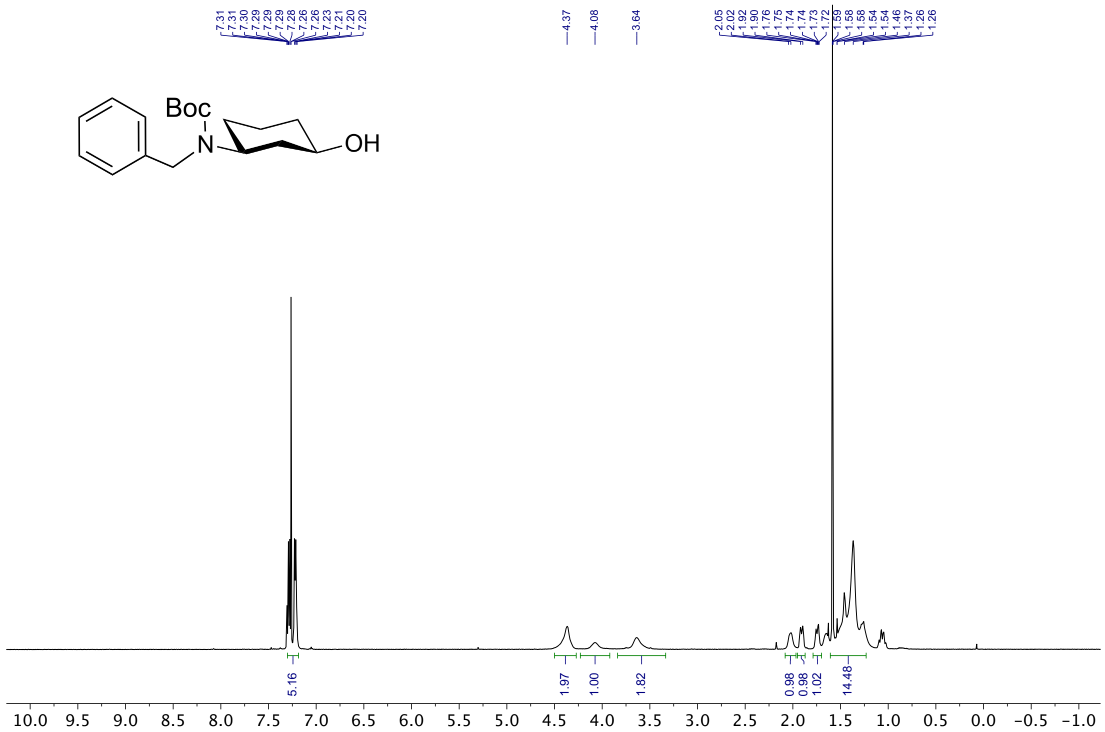

Figure S17. ^{1}H NMR (500 MHz, $CDCl_3$) of compound **S3**

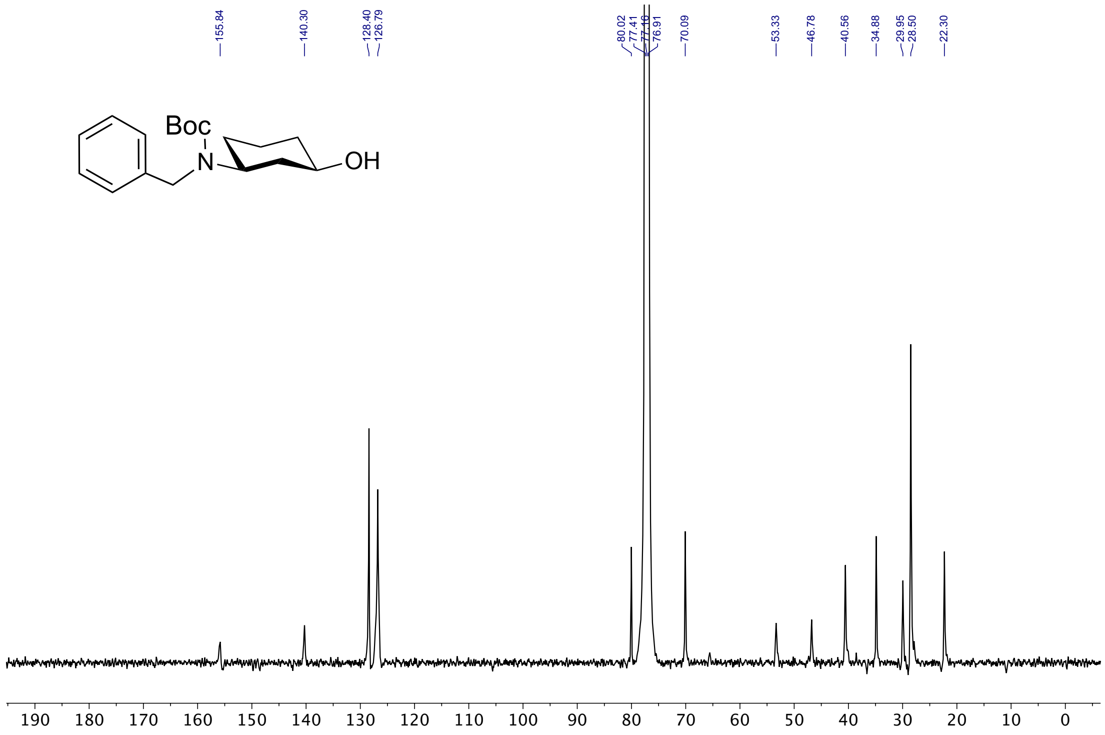

Figure S18. $^{13}C\{^{1}H\}$ NMR (126 MHz, $CDCl_3$) of compound **S3**

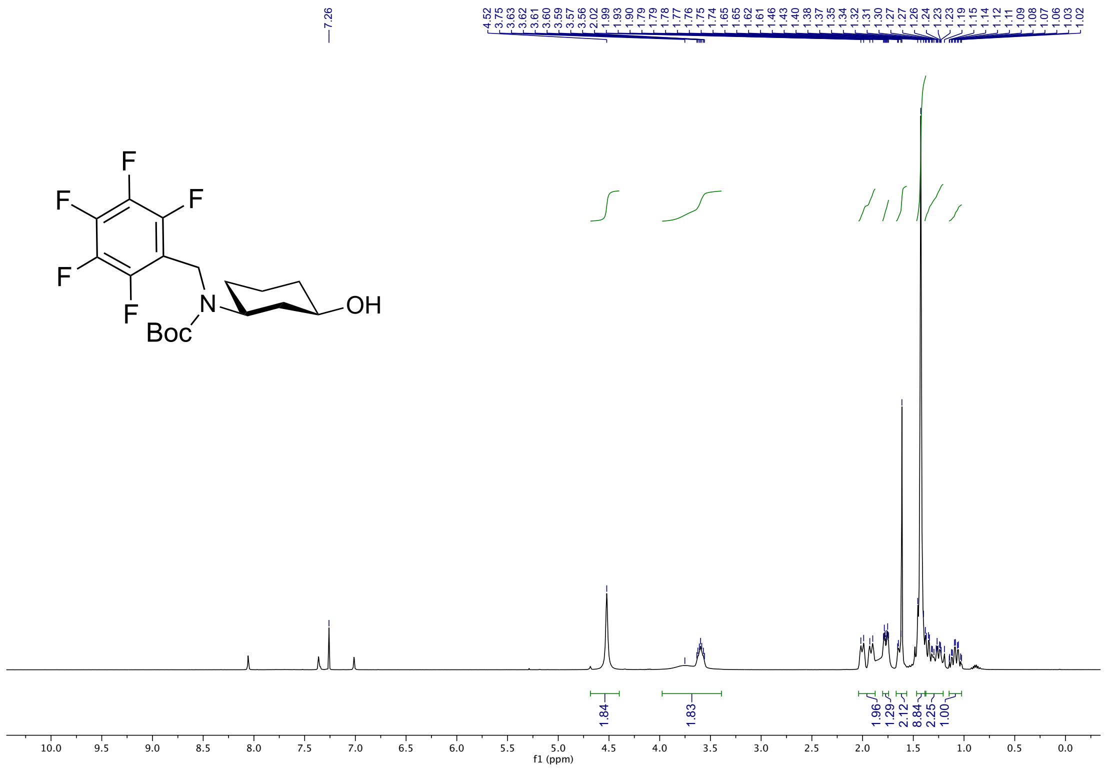

Figure S19. ^{1}H NMR (300 MHz, $CDCl_3$) of compound **S4**

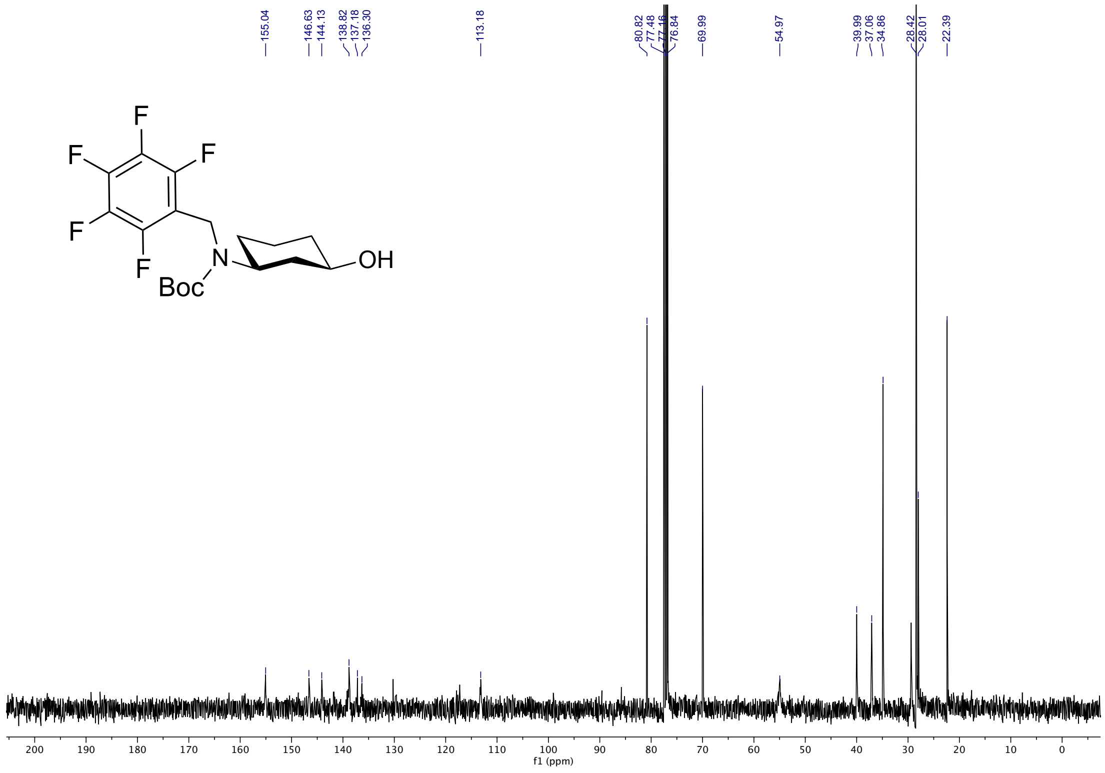

Figure S20. $^{13}C\{^{1}H\}$ NMR (75 MHz, $CDCl_3$) of compound **S4**

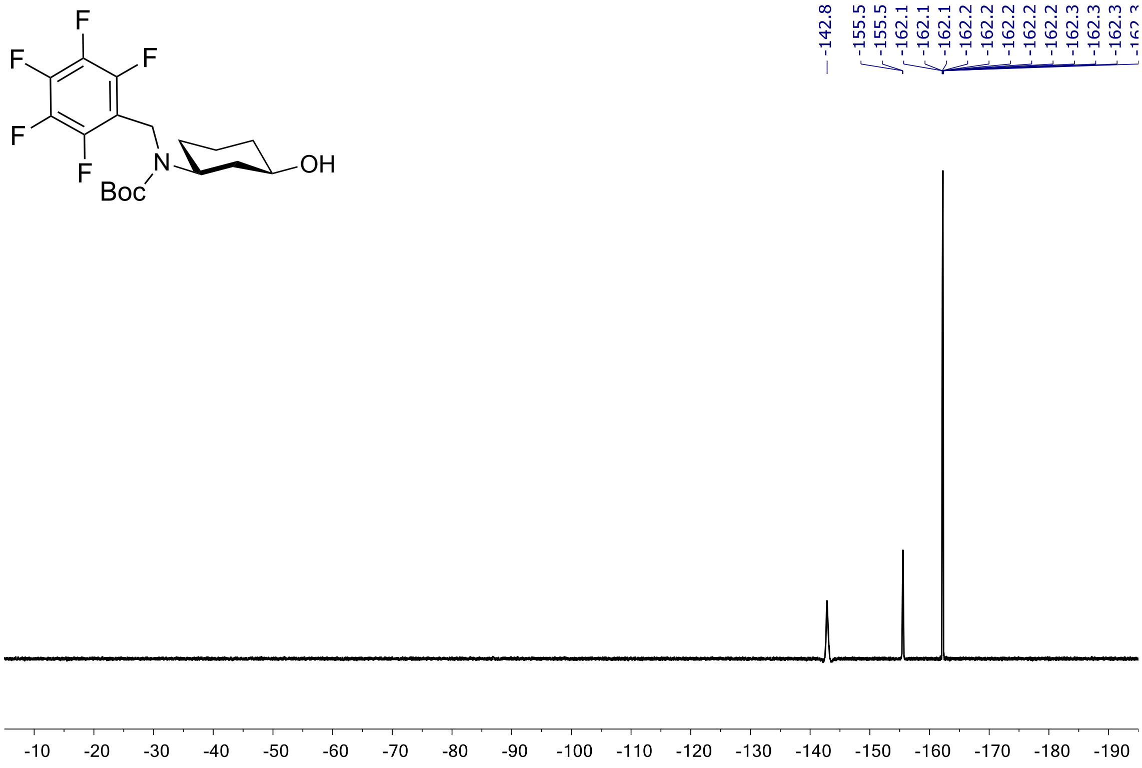

Figure S21. ^{19}F NMR (282 MHz, $CDCl_3$) of compound **S4**

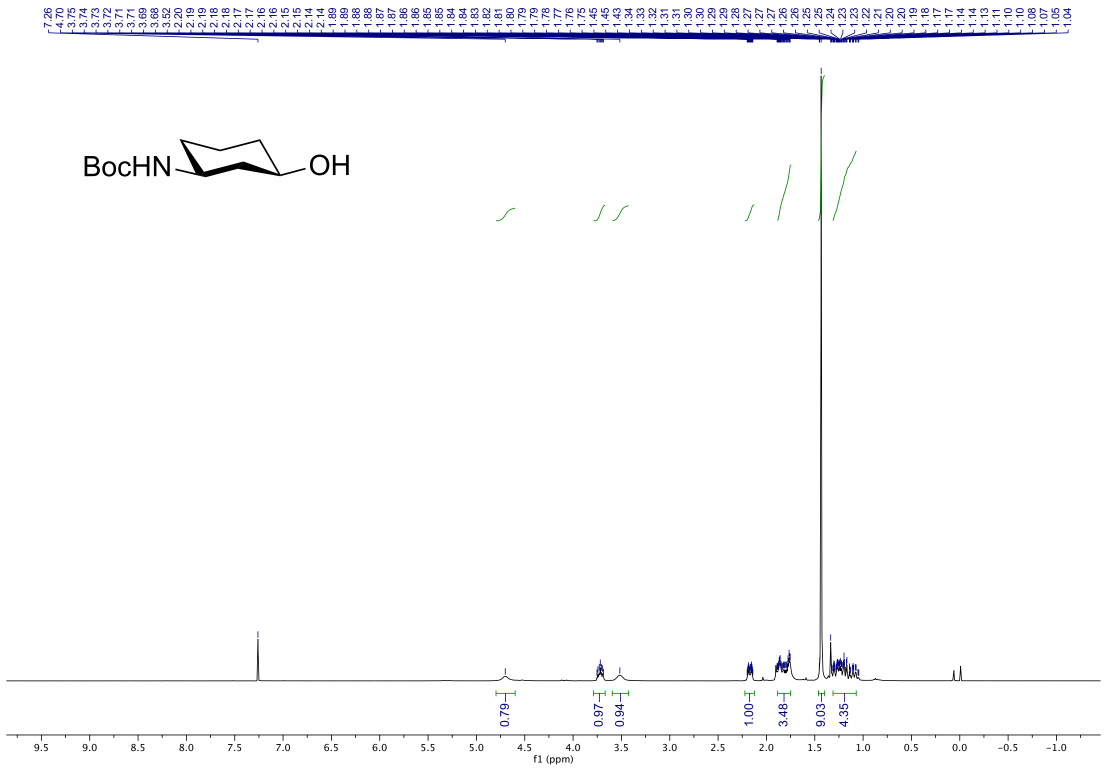

Figure S22. ^{1}H NMR (400 MHz, $CDCl_3$) of compound **S5**

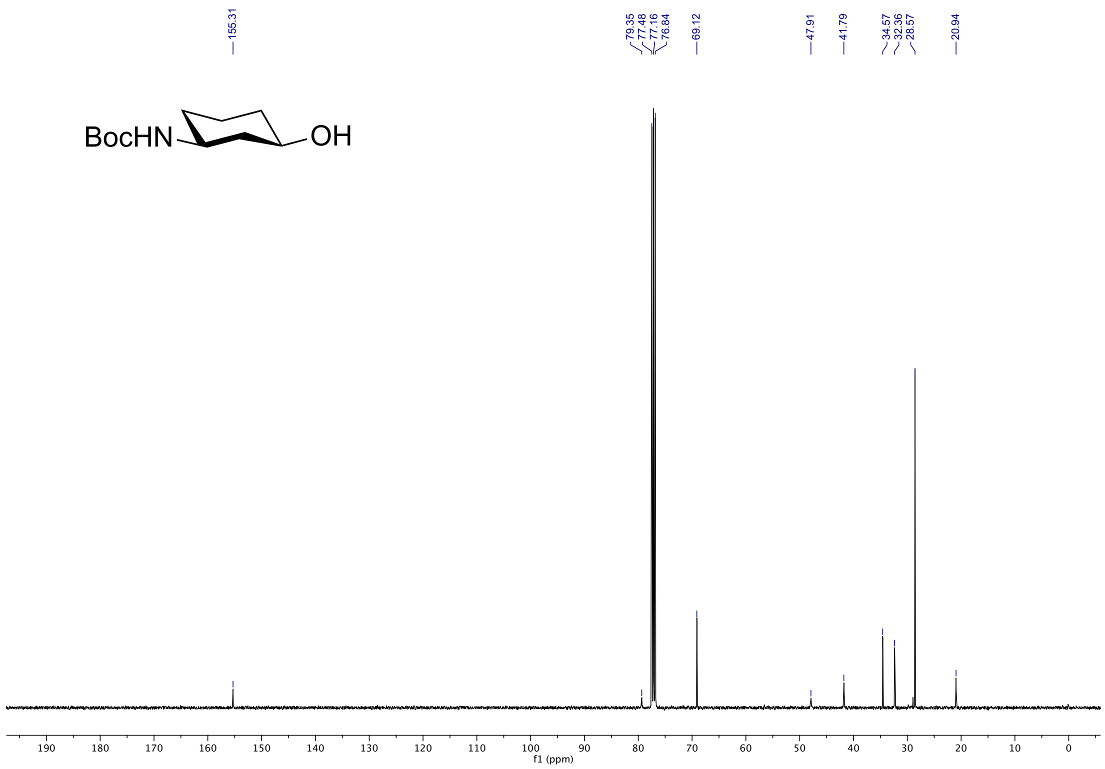

Figure S23. $^{13}C\{^{1}H\}$ NMR (100 MHz, $CDCl_3$) of compound **S5**

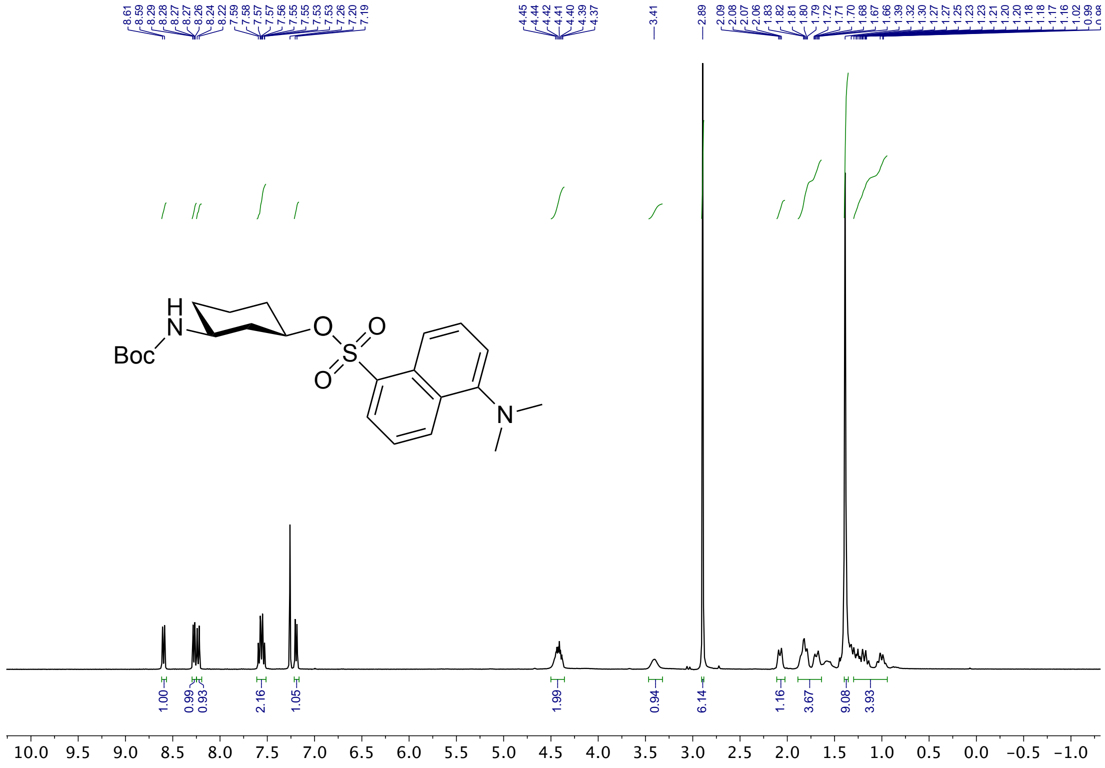

Figure S24. ^{1}H NMR (300 MHz, $CDCl_3$) of compound **S6**

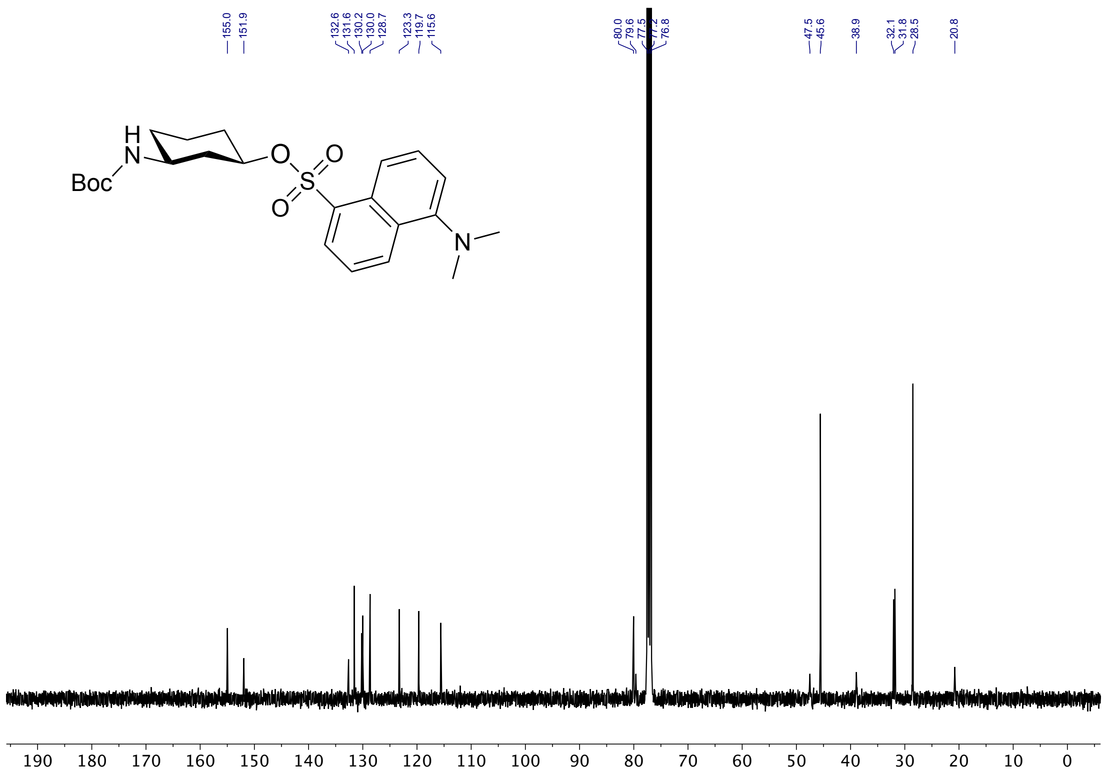

Figure S25. $^{13}C\{^1H\}$ NMR (75 MHz, $CDCl_3$) of compound **S6**

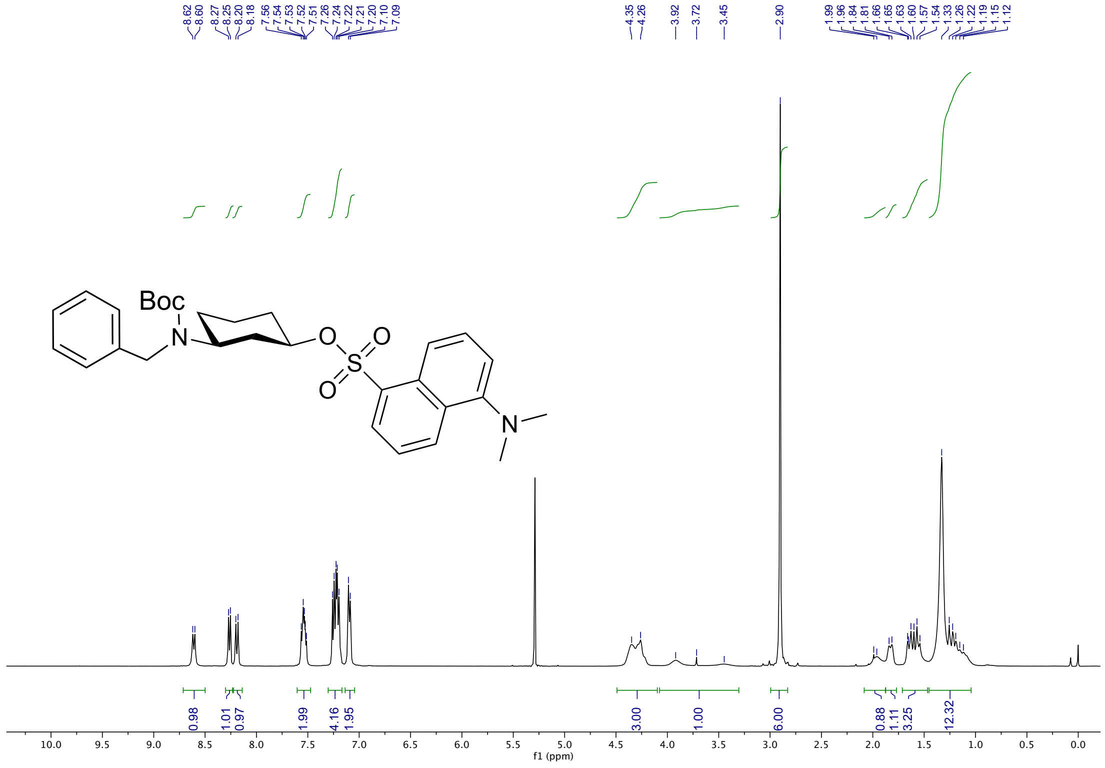

Figure S26. ^1H NMR (400 MHz, $CDCl_3$) of compound **S7**

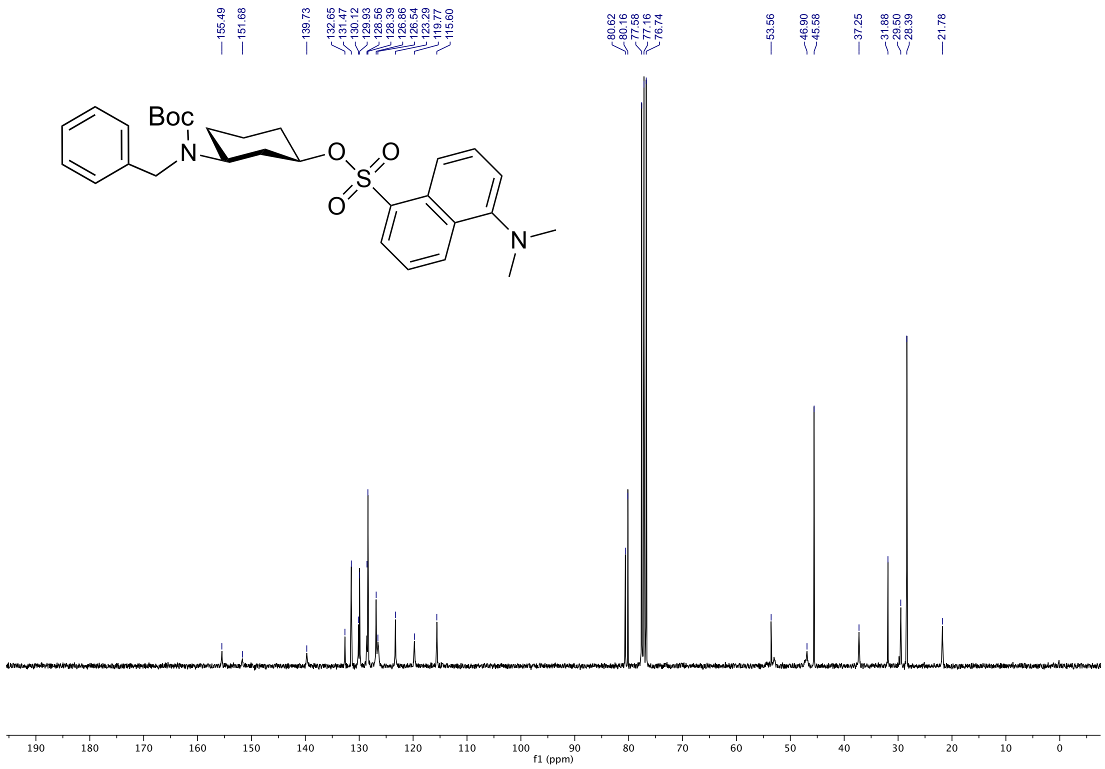

Figure S27. $^{13}C\{^{1}H\}$ NMR (100 MHz, $CDCl_3$) of compound **S7**

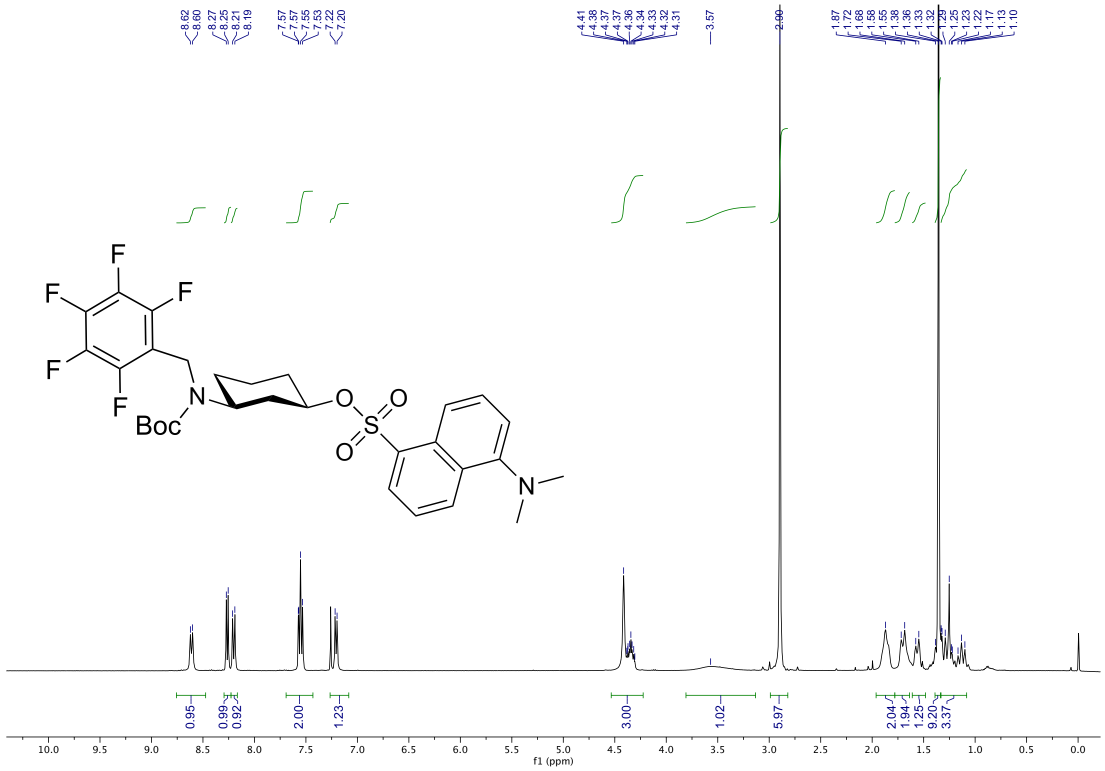

Figure S28. ^{1}H NMR (400 MHz, $CDCl_3$) of compound **3**

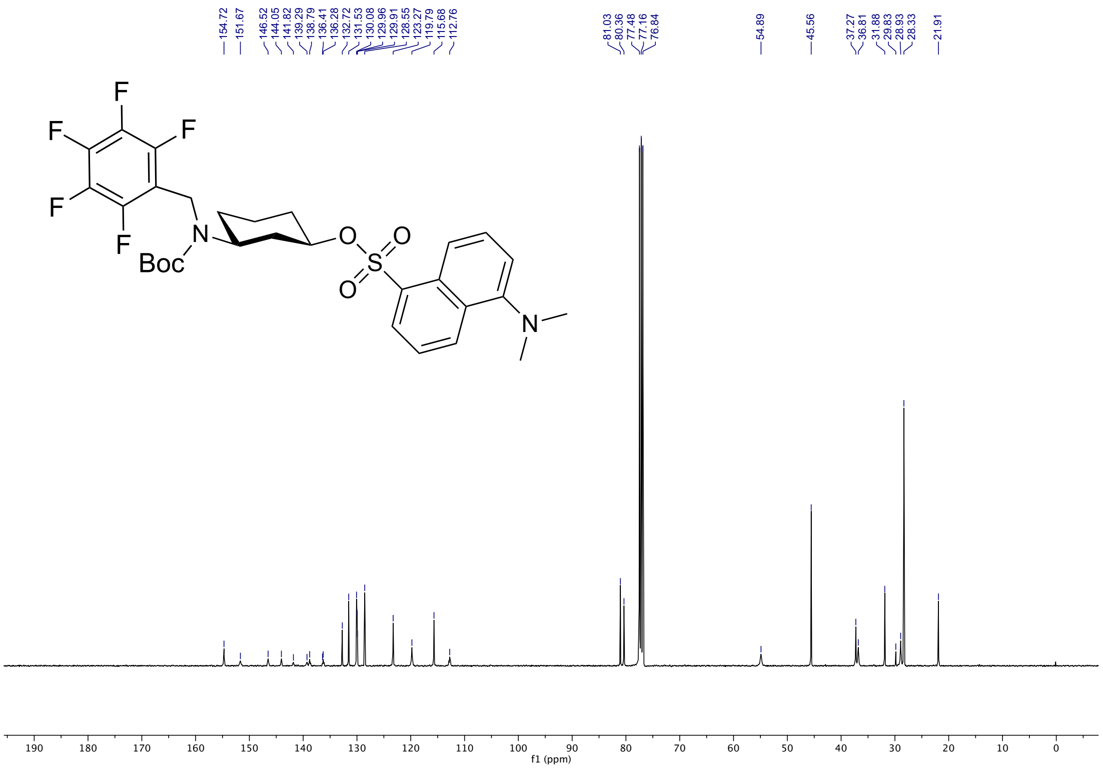

Figure S29. $^{13}C\{^{1}H\}$ NMR (100 MHz, $CDCl_3$) of compound **3**

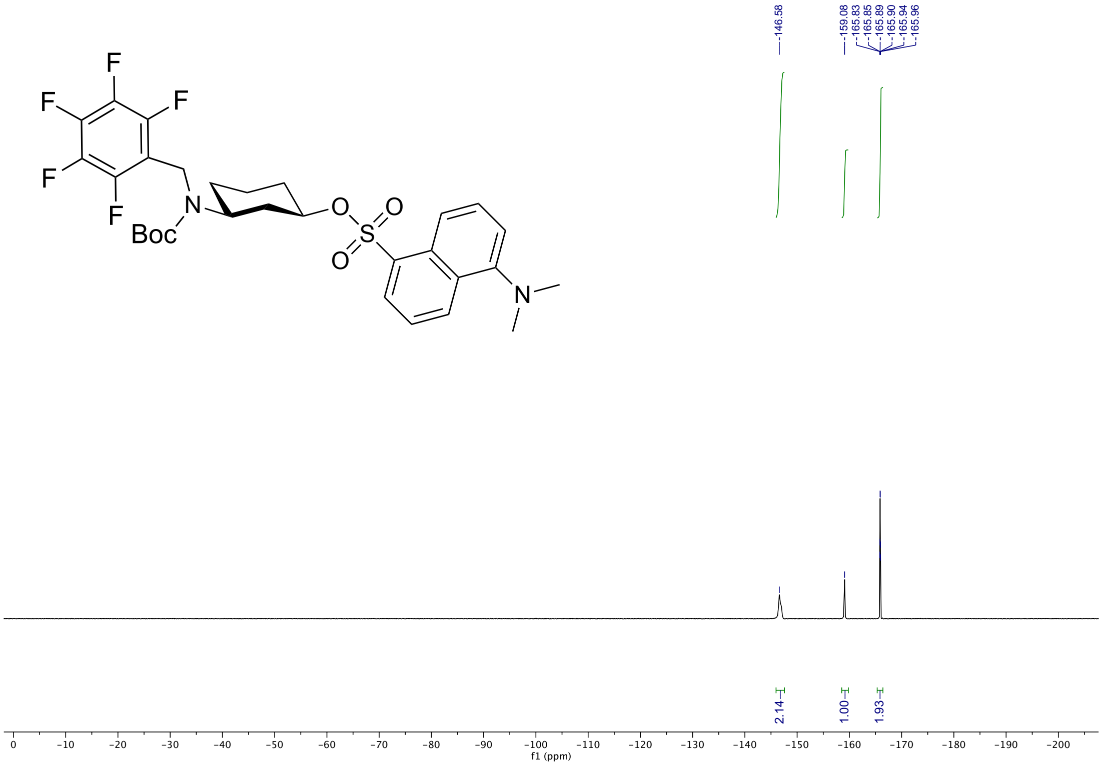

Figure S30. ^{19}F NMR (376 MHz, $CDCl_3$) of compound **3**

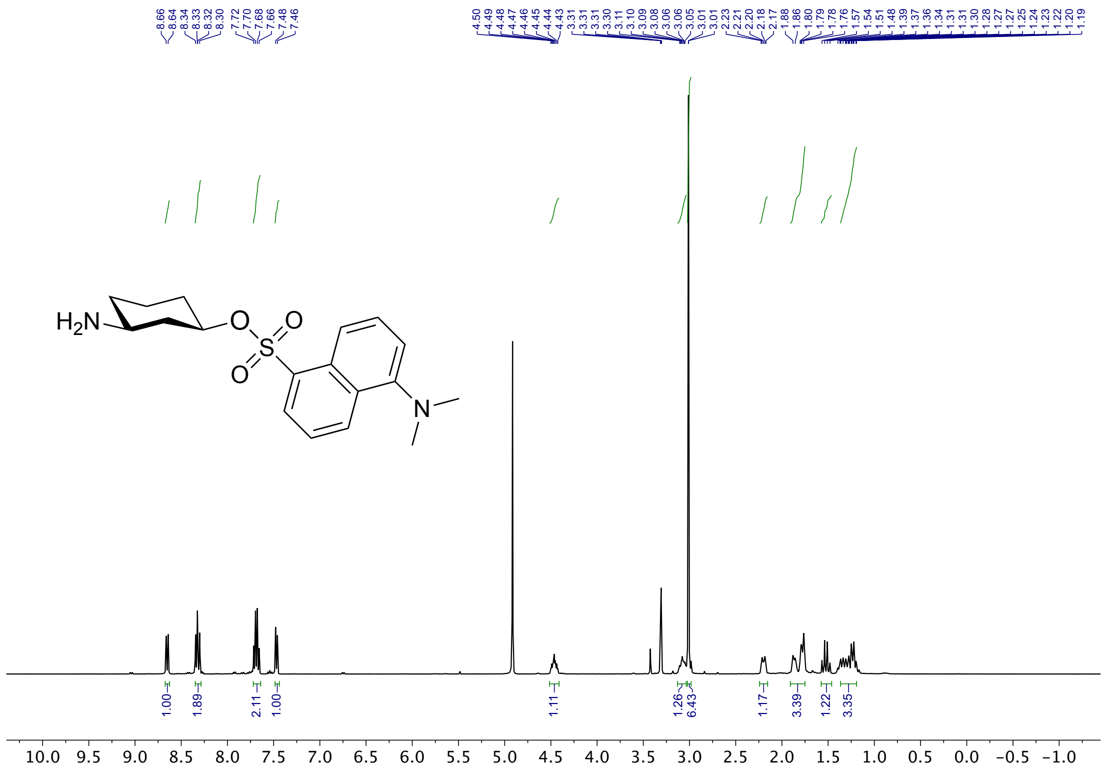

Figure S31. ^{1}H NMR (300 MHz, CD_3OD) of compound **2a**

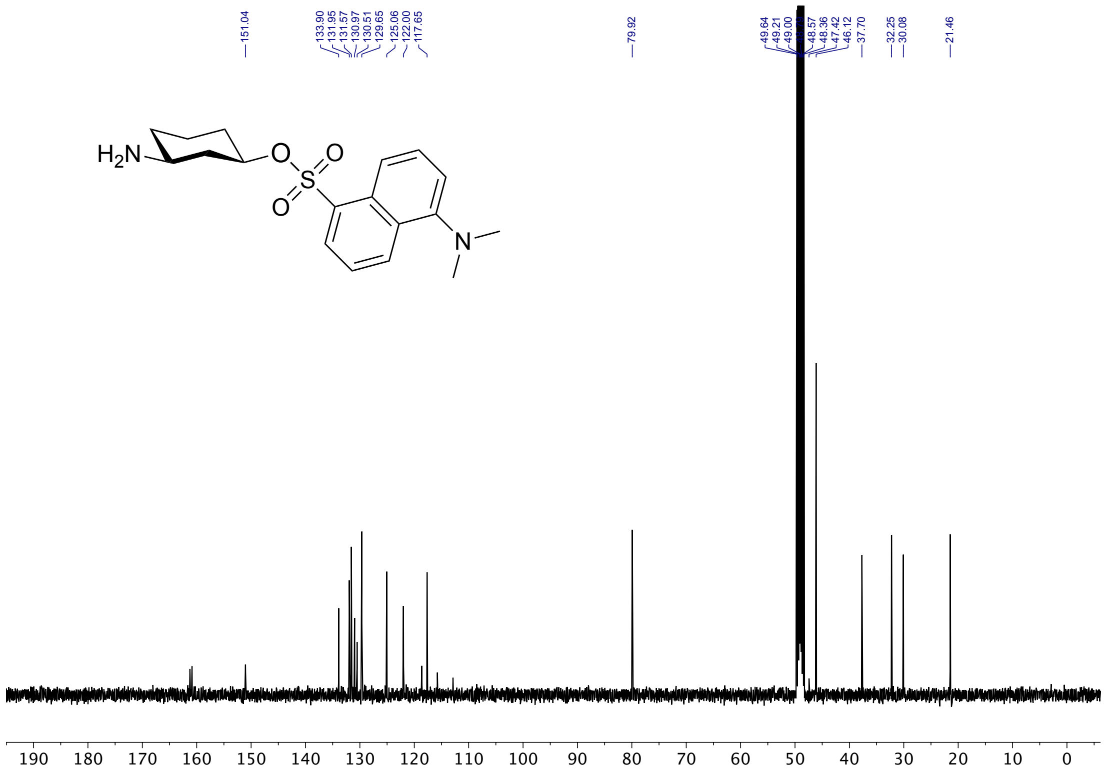

Figure S32. ^{13}C{^{1}H} NMR (75 MHz, CD_3OD) of compound **2a**

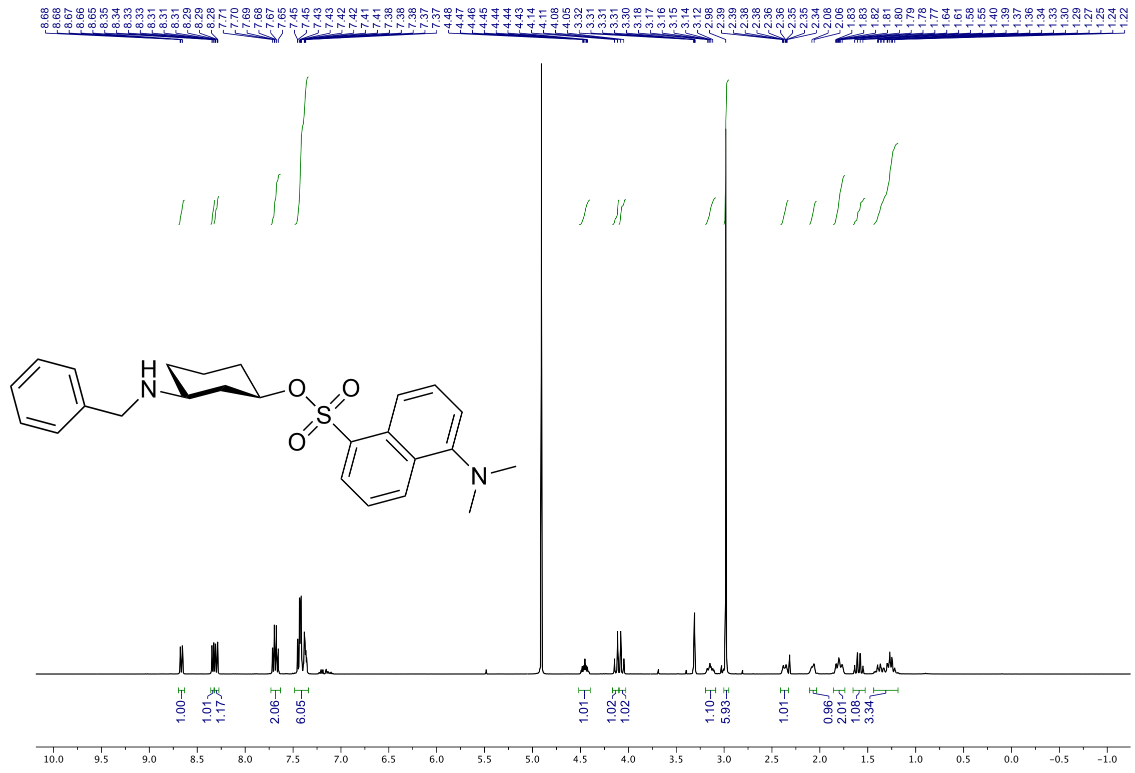

Figure S33. ^{1}H NMR (400 MHz, CD_3OD) of compound **2b**

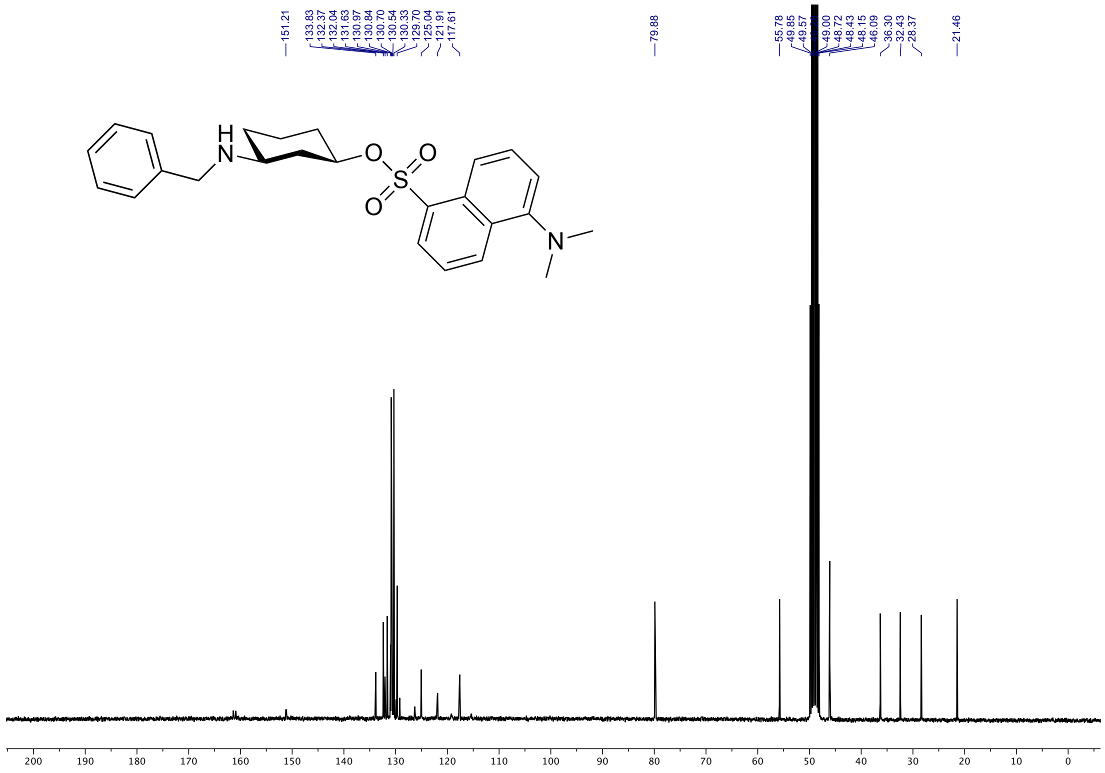

Figure S34. $^{13}C\{^{1}H\}$ NMR (100 MHz, CD_3OD) of compound **2b**

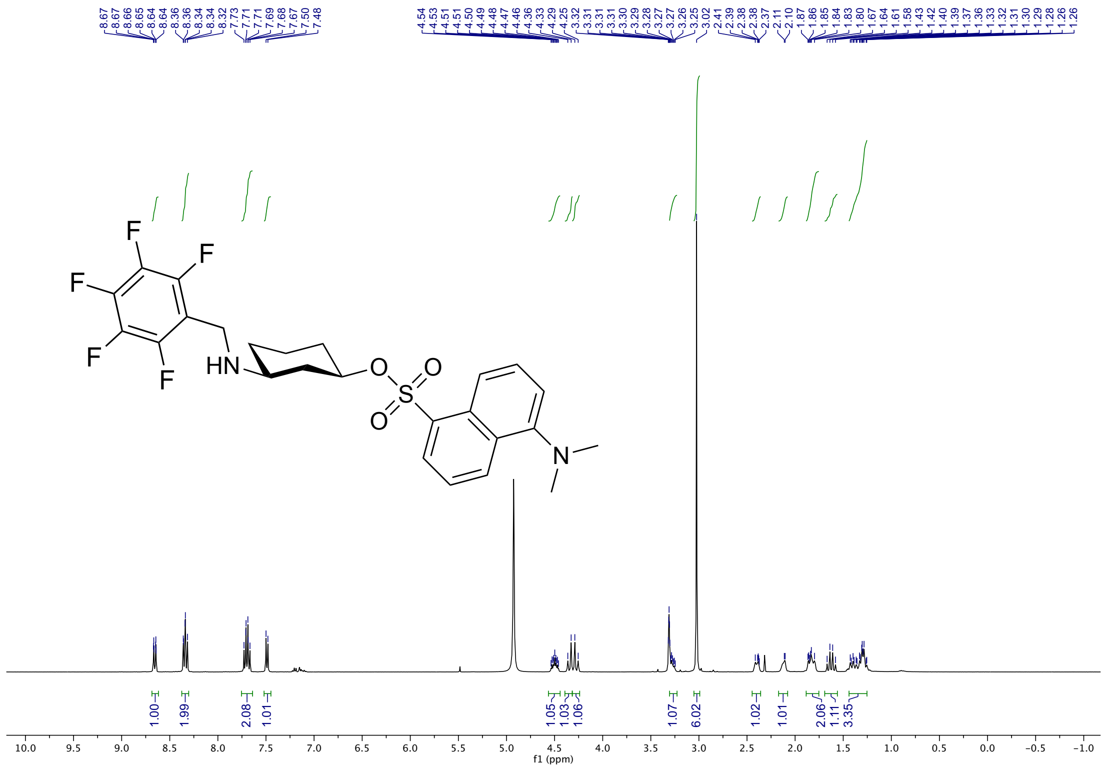

Figure S35. ^{1}H NMR (400 MHz, CD_3OD) of compound **2c**

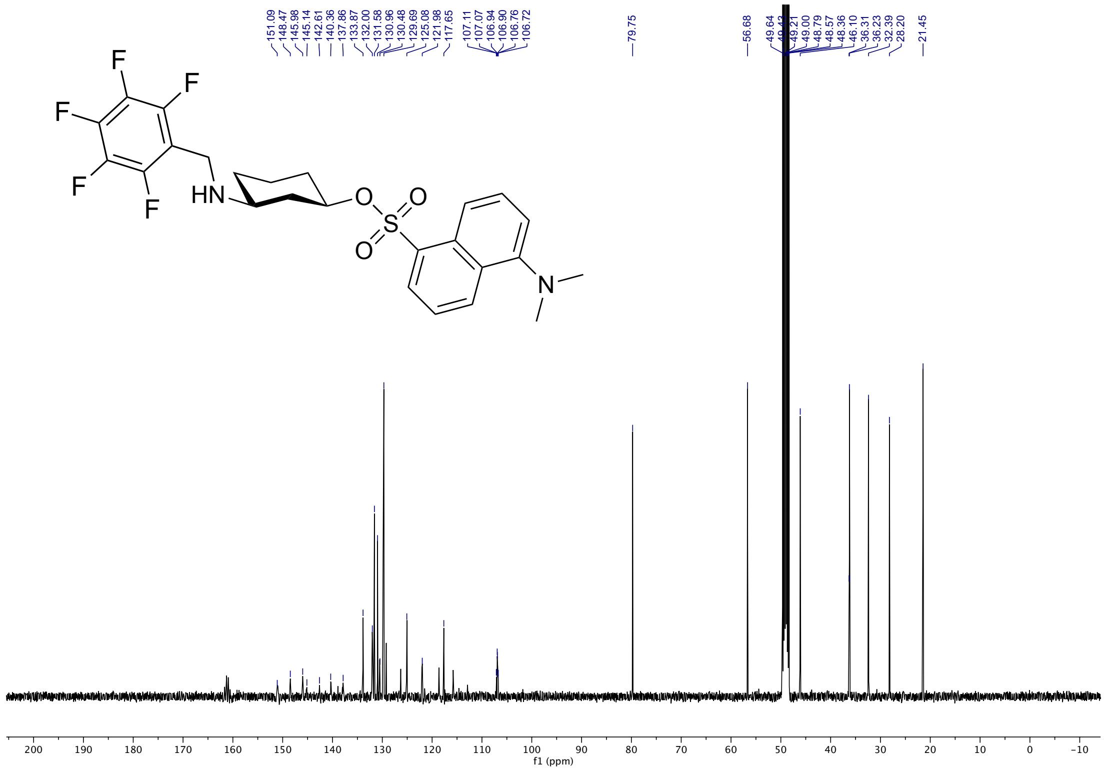

Figure S36. $^{13}C\{^{1}H\}$ NMR (100 MHz, CD_3OD) of compound **2c**

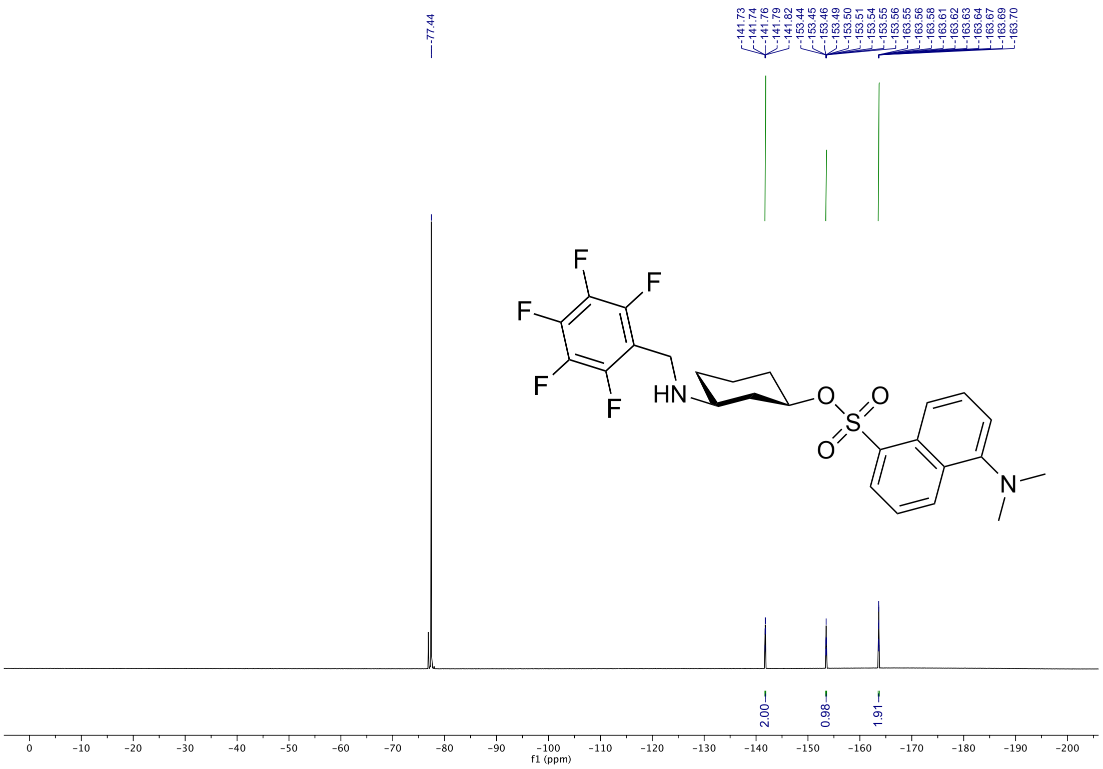

Figure S37. ^{19}F NMR (376 MHz, CD_3OD) of compound **2c**

11. References

(1) Friese, F. W.; Studer, A. Deoxygenative Borylation of Secondary and Tertiary Alcohols. *Angew. Chemie Int. Ed.* **2019**, *58*, 9561–9564.

(2) Nagase, T.; Takahashi, T.; Sasaki, T.; Nagumo, A.; Shimamura, K.; Miyamoto, Y.; Kitazawa, H.; Kanesaka, M.; Yoshimoto, R.; Aragane, K.; Tokita, S.; Sato, N. Synthesis and Biological Evaluation of a Novel 3-Sulfonyl-8-Azabicyclo[3.2.1]Octane Class of Long Chain Fatty Acid Elongase 6 (ELOVL6) Inhibitors. *J. Med. Chem.* **2009**, *52*, 4111–4114.
